# CRISPR Technology: From Bench to Bedside Applications

**DOI:** 10.1002/mco2.71011

**Published:** 2026-11-09

**Authors:** Xinzhi Ma, Lingyu Li, Yingli Sun

**Affiliations:** ^1^ Central Laboratory & Shenzhen Key Laboratory of Epigenetics and Precision Medicine For Cancers National Cancer Center/National Clinical Research Center for Cancer/Cancer Hospital & Shenzhen Hospital Chinese Academy of Medical Sciences and Peking Union Medical College Shenzhen China

**Keywords:** base editing, clinical translation, CRISPR‐Cas9, CRISPR delivery, gene therapy, genome editing, prime editing

## Abstract

CRISPR‐Cas genome editing represents one of the most transformative advances in modern molecular biology, enabling programmable site‐specific genetic modification that has revolutionized functional genomics and precision therapeutic development. Beyond canonical Cas9 nucleases, the CRISPR toolkit has diversified into precision base editors, prime editors, CRISPR‐associated transposases, and Cas12/Cas13 RNA‐targeting effectors, each with distinct mechanistic properties. Clinically, ex vivo CRISPR therapies for hemoglobinopathies have secured regulatory approval, while base editing, prime editing, and in vivo Chimeric Antigen Receptor (CAR)‐T candidates are advancing through trials for a broad spectrum of hematologic, oncologic, and rare genetic disorders. However, existing reviews largely address technical innovation or clinical translation in isolation, lacking a holistic, up‐to‐date synthesis of the full bench‐to‐bedside trajectory of CRISPR technology. This review systematically dissects the molecular mechanisms, advantages, and limitations of core CRISPR editing platforms; summarizes pivotal clinical data across hemoglobinopathies, CAR‐T immunotherapy, muscular dystrophy, cystic fibrosis, cardiometabolic diseases and rare monogenic diseases; evaluates delivery systems including lipid nanoparticles and viral vectors; and discusses key translational barriers such as off‐target genotoxicity and immunogenicity alongside emerging optimization strategies. This work provides a holistic bench‐to‐bedside framework for CRISPR research, offering critical insights to advance next‐generation precision genetic medicine.

## Graphical abstract

This review systematically dissects the molecular mechanisms, advantages, and limitations of core CRISPR editing platforms including base editors (Panel A) and prime editing (Panel B); summarizes pivotal clinical data across hemoglobinopathies, CAR‐T immunotherapy, muscular dystrophy, cystic fibrosis, cardiometabolic diseases, and rare monogenic diseases; evaluates delivery systems including lipid nanoparticles and viral vectors; and discusses key translational barriers such as off‐target genotoxicity and immunogenicity alongside emerging optimization strategies. This work provides a holistic bench‐to‐bedside framework for CRISPR research, offering critical insights to advance next‐generation precision genetic medicine.

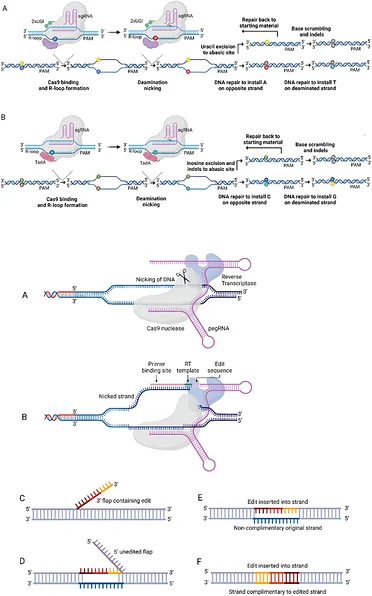

## 1 Introduction

Precise manipulation of the genetic code in living organisms stands as one of the most transformative breakthroughs in modern life sciences, holding unprecedented promise for dissecting fundamental biological mechanisms, engineering cellular behaviors, and correcting pathogenic mutations. Before the emergence of CRISPR technology, scientists mainly used zinc finger nucleases (ZFNs) and transcription activator‐like effector nucleases (TALENs) as the primary platforms for genome editing [1, 2]. Both systems rely on chimeric protein architectures, in which a programmable DNA‐binding domain is fused to the non‐specific DNA cleavage domain of the FokI restriction endonuclease.

ZFNs achieve DNA recognition through tandem arrays of zinc finger motifs, with each individual module typically recognizing a ∼3 bp nucleotide triplet. A significant limitation of ZFNs is the context‐dependent nature of this recognition, where the binding affinity and specificity of a given finger can be influenced by its neighboring fingers. As a result, ZFN engineering has historically been costly, labor‐intensive, and prone to off‐target cleavage events due to inherent limitations in targeting specificity [3].

TALENs, by contrast, exploit a far more modular DNA recognition mechanism. Their DNA‐binding domain consists of tandem repeats, each of which recognizes a single base pair through two hypervariable amino acids known as repeat‐variable diresidues (RVDs). This one‐repeat‐to‐one‐base‐pair code substantially simplified construct design and conferred superior targeting flexibility relative to ZFNs. However, the requirement to assemble large, highly repetitive TALEN expression constructs has remained a practical barrier to their broad and routine adoption (Figure 1).

**FIGURE 1:**
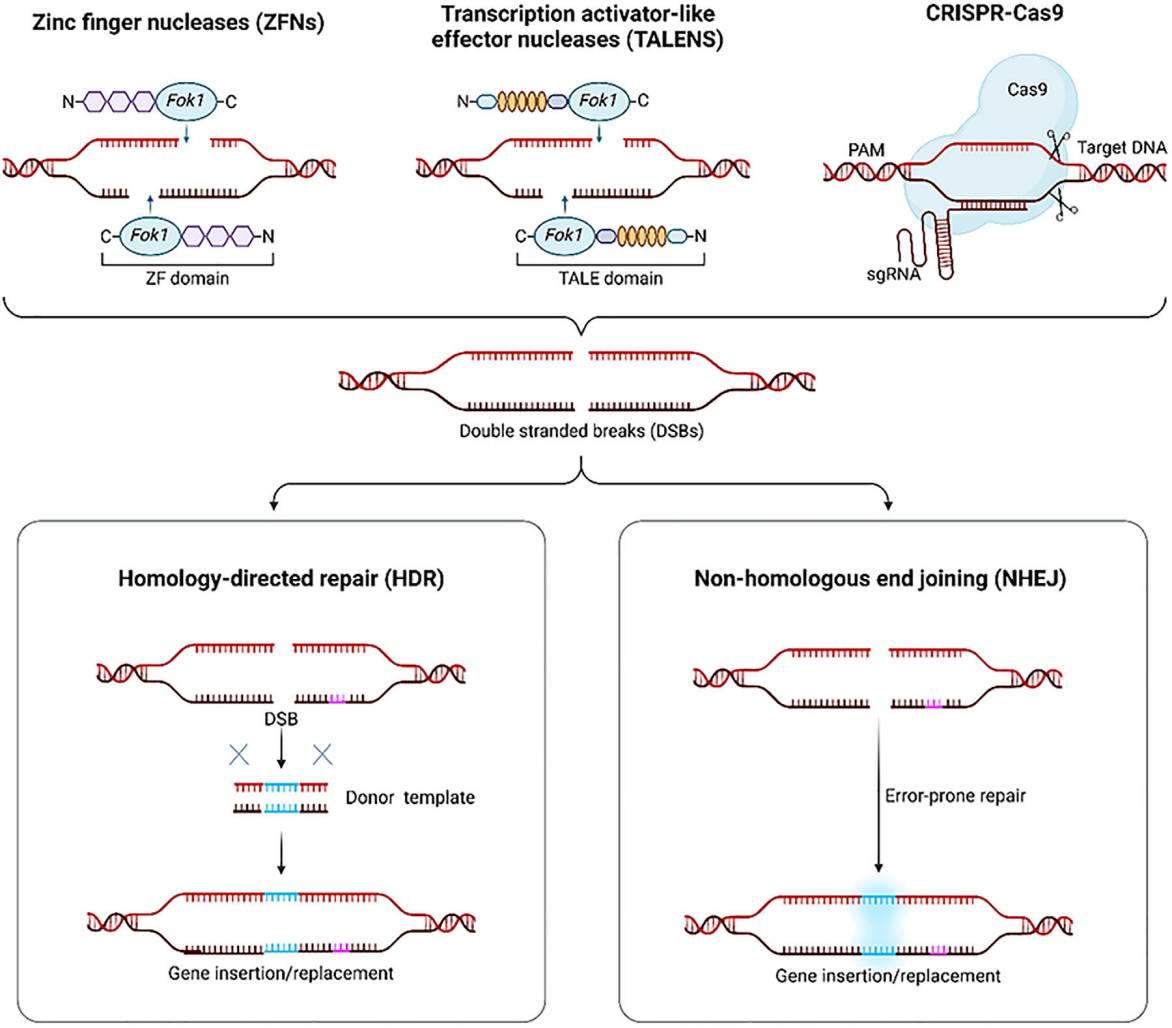
Three major genome editing platforms and the downstream DNA double‐strand break (DSB) repair pathways. ZFNs and TALENs are composed of tandem DNA‐binding domains fused to a FokI nuclease domain. Since FokI requires dimerization to exert endonuclease activity, two monomers must bind to opposite strands of the target DNA with proper spacing to induce DSBs. Both systems rely on protein‐DNA interactions for target recognition and require no auxiliary DNA motifs for cleavage. In contrast, the CRISPR‐Cas9 system achieves DNA targeting via RNA‐DNA base complementarity. It consists of the Cas9 endonuclease and a single guide RNA (sgRNA), which directs Cas9 to target sequences through Watson–Crick base pairing. Cas9 binding and cleavage are strictly dependent on the protospacer adjacent motif (PAM): a short conserved sequence located immediately at the 3’ end of the sgRNA‐targeted DNA strand. As an absolute prerequisite for Cas9 function, PAM acts as the initial binding anchor, enables strand invasion and sgRNA‐DNA hybridization, and is the mandatory element that allows Cas9 to distinguish target DNA from non‐target genomic sequences. Following site‐specific DSB generation, two major endogenous cellular DNA repair pathways are activated to mediate genome modification. The high‐fidelity homology‐directed repair (HDR) pathway uses an exogenous homologous donor DNA template to achieve precise, user‐defined gene insertion or replacement at the DSB site. The error‐prone non‐homologous end joining (NHEJ) pathway directly ligates broken DNA ends without a homologous template; it typically introduces random insertions or deletions (indels) to disrupt target gene function, and can also mediate gene insertion or replacement in specific contexts.

The advent of CRISPR‐Cas systems has revolutionized the genome editing landscape by overcoming many of these earlier technical constraints. A set of uncharacterized tandem repeat sequences in *Escherichia coli* was first identified in 1987 [4]. The term “Clustered Regularly Interspaced Short Palindromic Repeats (CRISPR)” was officially named by Jansen et al. in 2002 to describe the family of clustered regularly interspaced short palindromic repeats in prokaryotic genomes [5]. A pivotal functional breakthrough came in 2007, when Barrangou et al. [6] demonstrated that prokaryotic CRISPR‐Cas systems function as adaptive genome defense mechanisms that recognize and target foreign nucleic acids from viruses (phages); these sequences are which are integrated as novel “spacers” into the host's CRISPR genomic loci. These loci are transcribed as long precursor transcripts (pre‐crRNA) and undergo precise processing to generate mature CRISPR RNAs (crRNAs). crRNAs serve as molecular guides, directing Cas proteins to identify and destroy complementary invasive nucleic acids.

Subsequent investigations identified that the Type II CRISPR‐Cas system can specifically cleave phage DNA [7], an activity which was found to depend on the Cas9 protein [8] and trans‐activating crRNA (tracrRNA), which is essential for the maturation of the crRNA [9]. A landmark biochemical characterization in 2012 definitively established Cas9 as an RNA‐guided DNA endonuclease, whose targeting specificity is dictated by a dual‐RNA complex comprising both crRNA and tracrRNA [10, 11].The Cas9 nuclease identifies its genomic target through two critical requirements: base‐pairing between the guide RNA's spacer sequence and the target DNA strand, and the presence of a short protospacer adjacent motif (PAM) near the target site. After target binding, Cas9 catalyzes DNA cleavage, generating a double‐strand break (DSB). DSB repair via cellular DNA repair pathways results in genetic editing. Error‐prone non‐homologous end joining (NHEJ) often results in small insertions or deletions (indels), while the provision of an exogenous DNA template can engage the more precise homology‐directed repair (HDR) pathway to introduce specific genetic modifications (Figure 1). The system was significantly simplified by integrating crRNA and tracrRNA structure into a single‐guide RNA (sgRNA), creating a versatile, programmable platform with a one‐nuclease‐to‐one‐guide design. This innovation paved the way for programmable genome editing, whereby targeting Cas9 to any genomic locus simply requires the design of a complementary sgRNA. In 2013, the groundbreaking work of Cong et al., Mali et al., and Jinek et al. first successfully applied the CRISPR/Cas9 system to genome editing in human cells [12, 13, 14]. Multiple laboratories demonstrated the gene editing ability of CRISPR‐Cas9 in a wide range of biological models, including yeast, nematodes, fruit flies, and mammalian cells [12, 13, 15, 16, 17, 18, 19]. These groundbreaking studies collectively established the enormous potential of the CRISPR‐Cas9 system in gene function research and genome editing [20, 21]. Subsequently, CRISPR‐Cas9 technology has been widely applied in functional genomics, genetic disease research, and applied research in agriculture and biomedicine, laying a solid foundation for the advancement of genome editing technology [22, 23, 24, 25].

This foundational discovery rapidly democratized genome editing and the toolkit has since evolved: base editors enable single‐nucleotide conversions [26, 27] without DSBs; prime editing (PE) allows versatile DNA rewriting, including insertions, deletions, and replacements with minimal byproducts [28]; CRISPR interference (CRISPRi) and CRISPR activation (CRISPRa) provide reversible, tunable transcriptional regulation without altering the genomic sequence, facilitating functional genomics studies [29]; multi‐gene editing permits simultaneous modification of several loci, streamlining complex genetic engineering; transposase‑directed systems enable efficient, targeted integration of large DNA fragments, expanding the scope of synthetic biology [30]; and Cas12 and Cas13 systems extend targeting to AT‐rich genomic regions and RNA transcripts, respectively, broadening the utility of CRISPR in genome‐wide screens, antiviral strategies, and transcriptional modulation [31, 32, 33].

In parallel with technological innovation, clinical translation of CRISPR‐based therapies has accelerated dramatically in recent years. Ex vivo CRISPR‐edited therapies for sickle cell disease (SCD) and β‐thalassemia have received regulatory approval, while multiple base editing (BE) and PE candidates have entered clinical trials for hematologic, cardiometabolic, and rare genetic disorders, alongside burgeoning applications in Chimeric Antigen Receptor (CAR)‐T cell engineering.

Despite these remarkable advances, a comprehensive, up‐to‐date synthesis that systematically maps the full “bench‐to‐bedside” trajectory of CRISPR technology remains lacking. This review provides a systematic overview of CRISPR technology from fundamental research to clinical translation. We first dissect the molecular mechanisms, advantages, and limitations of core editing platforms spanning base editors, prime editors, transposase systems, and RNA‐targeting tools. We then summarize the clinical landscape of leading CRISPR‐based therapies, with detailed analysis of pivotal trial data across hemoglobinopathies, CAR‐T immunotherapy, muscular dystrophy, cystic fibrosis (CF), and cardiometabolic diseases. Next, we examine state‐of‐the‐art delivery systems and tissue‐specific targeting strategies. Finally, we discuss key translational challenges and highlight emerging solutions, offering a forward‐looking perspective on the future of CRISPR‐based precision medicine.

## 2 Genome Editing Platforms for Translational Research

The CRISPR toolkit has expanded far beyond canonical Cas9 nucleases into a diversified array of editing modalities, each with distinct mechanistic features and translational profiles. Nuclease‐dependent systems such as Cas9 and Cas12 enable robust gene disruption and form the technical foundation of first‐generation approved therapies, yet their reliance on DSBs carries inherent genotoxic risks. Base editors and prime editors achieve precise nucleotide alterations without DSBs, substantially improving safety for in vivo applications and expanding the scope of treatable pathogenic mutations. Complementing these, CRISPR‐based transcriptional regulators offer reversible gene expression control, and CRISPR‐associated transposases enable efficient large‐fragment genomic integration. This section evaluates each core platform from a translational perspective, summarizing its mechanism, therapeutic advantages, and remaining limitations to provide a technical framework for subsequent clinical discussion.

### 2.1 Base Editors

To overcome the inherent limitations of DSB‐dependent genome editing, BE technologies have been developed as a transformative class of precision genome editing tools that enable site‐specific single‐nucleotide modifications without inducing DSBs or requiring exogenous donor templates. The two most widely used canonical BE systems are cytosine base editors (CBEs) and adenine base editors (ABEs).

CBEs, first reported by Komor et al. in 2016 [27], are typically composed of a Cas9 nickase (nCas9, a Cas9 variant that only generates single‐strand DNA nicks rather than DSBs) fused to a cytidine deaminase domain and two tandem copies of uracil DNA glycosylase inhibitor (UGI). Guided by a single guide RNA (sgRNA), the complex localizes to the target genomic locus via PAM recognition, forms an R‐loop structure to expose single‐stranded DNA (ssDNA), and catalyzes the deamination of target cytosine (C) to uracil (U). The UGI moiety blocks cellular uracil excision repair to stabilize the U:G mismatch, and the nick on the non‐edited strand biases cellular repair machinery to use the deaminated strand as a template, ultimately achieving precise C•G to T•A conversion (Figure 2A).

**FIGURE 2:**
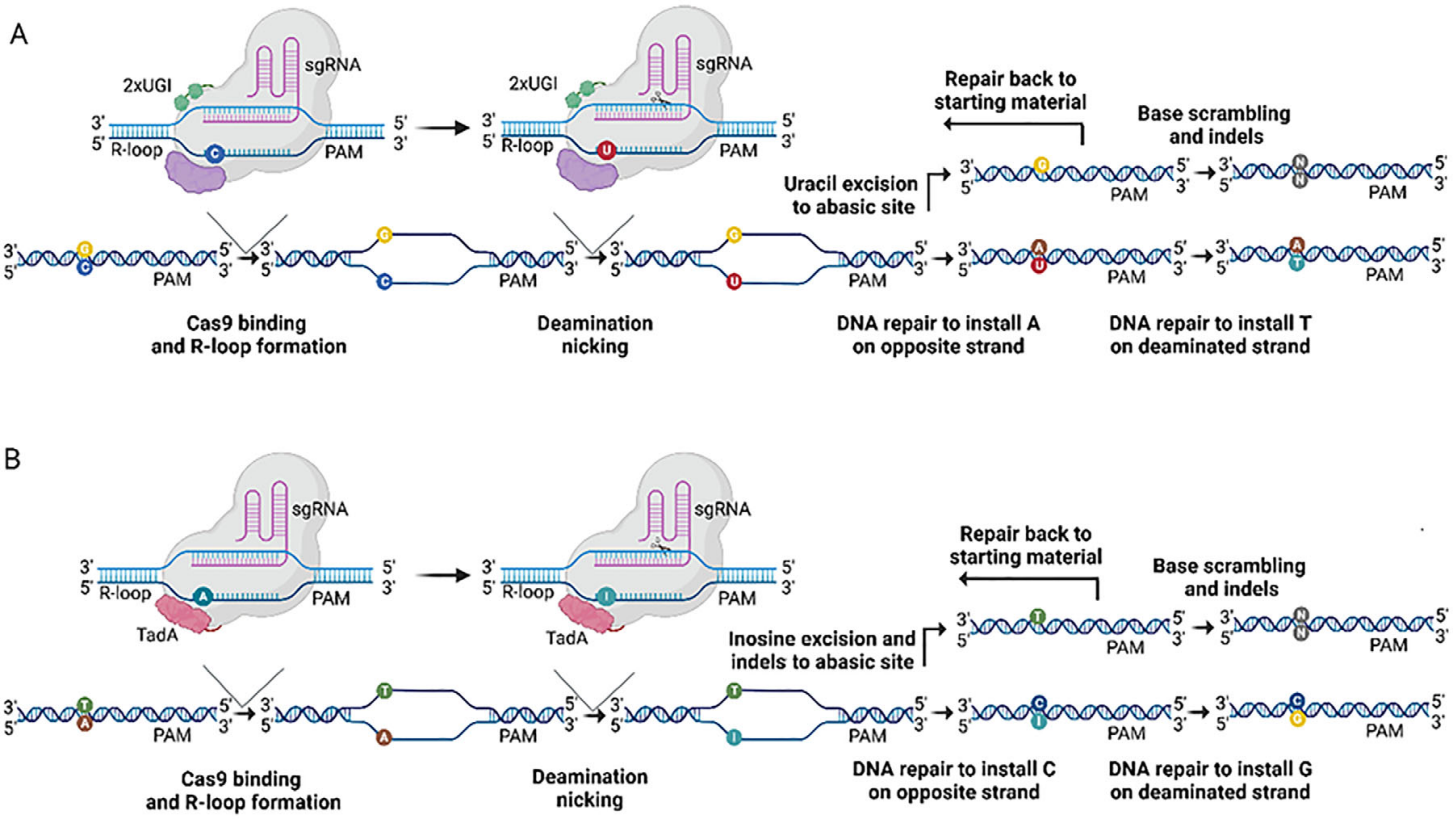
Molecular mechanisms of two base editing systems: cytosine base editor (CBE) and adenine base editor (ABE). Base editors are programmable genome editing tools that enable precise single‐nucleotide modifications without inducing DNA double‐strand breaks (DSBs). The molecular mechanisms of two canonical systems are annotated below. Panel A: Mechanism of the cytosine base editor (CBE) system. The CBE complex consists of three core functional modules: (1) Cas9 nickase (nCas9), a Cas9 variant with an inactivated RuvC domain that generates only single‐strand nicks (no DSBs) and serves as the DNA‐targeting scaffold; (2) a cytidine deaminase catalytic domain; and (3) 2 × UGI, a uracil DNA glycosylase inhibitor that suppresses cellular UNG activity to reduce unwanted DNA repair events. Guided by a single guide RNA (sgRNA), the complex is recruited to target loci via sequence complementarity. First, the nCas9‐sgRNA complex anchors to target DNA in a strictly protospacer adjacent motif (PAM)‐dependent manner; sgRNA‐DNA base pairing displaces the non‐complementary strand to form an R‐loop, with the exposed single‐stranded DNA (ssDNA) acting as the deaminase substrate. The cytidine deaminase then converts target cytosine (C) to uracil (U) within the R‐loop, while nCas9 introduces a nick on the non‐edited complementary strand to bias cellular repair toward the deaminated strand. The resulting U:G mismatch is resolved by endogenous repair pathways, yielding three outcomes: precise on‐target editing: permanent site‐specific C•G → T•A base pair conversion (intended outcome); reversion to unedited state: residual UNG excises uracil to form an abasic site, which is repaired back to the original C•G via base excision repair (BER), resulting in no editing; undesired byproducts: error‐prone repair of abasic sites causes base scrambling or insertions/deletions (indels), the major source of imprecise editing in CBE. Panel B: Mechanism of the adenine base editor (ABE) system. The ABE complex comprises the same nCas9 targeting scaffold and an engineered *E. coli* TadA adenosine deaminase evolved to act on ssDNA substrates. Targeting is also sgRNA‐guided and PAM‐dependent, with R‐loop formation consistent with the CBE system. The TadA domain catalyzes deamination of target adenine (A) to inosine (I), which is recognized as guanine (G) by cellular polymerases and repair machinery; nCas9 simultaneously nicks the non‐edited strand to bias repair. The resulting I:T mismatch is resolved by endogenous pathways, with three parallel outcomes: precise on‐target editing: permanent site‐specific A•T → G•C base pair conversion (intended outcome); reversion to unedited state: cellular inosine excision enzymes remove inosine, and BER restores the original A•T base pair; undesired byproducts: error‐prone repair leads to base scrambling or indels, representing the imprecise editing limitations of ABE.

Subsequently, Gaudelli et al. developed ABEs in 2017 [26], expanding the scope of BE to enable A•T to G•C conversion. The canonical ABE system consists of an nCas9 scaffold fused to an engineered tRNA‐specific adenosine deaminase (TadA), which catalyzes the deamination of target adenine (A) to inosine (I). Inosine is recognized as guanine (G) by cellular polymerases and repair machinery, and similarly, the strand‐specific nick drives the cellular repair pathway to complete the permanent A•T to G•C base pair conversion (Figure 2B).

Despite their high precision, both CBE and ABE systems can produce undesired byproducts, including base scrambling and unintended insertions and deletions (indels), due to incomplete inhibition of endogenous base excision repair or error‐prone repair of intermediate abasic sites, which remain key limitations to be addressed for clinical translation.

Recent advances in BE technology have focused on two core challenges: improving editing precision and expanding the scope of editable mutations. To improve editing precision, Perrotta et al. combined engineered guide RNA screening with phage‐assisted directed evolution to develop high‐fidelity variants such as ABE8e‐V28C, which enhanced editing precision by several‐fold and demonstrated exceptional perfect correction rates in a therapeutic model of Parkinson's disease [34]. For expanding the scope of editable mutations, Ye et al. broke away from the conventional deaminase‐dependent framework by developing deaminase‐free base editors (DAF‐BEs) based on engineered DNA glycosylases [35]. These editors achieve efficient C‐to‐G and T‐to‐G transversion editing, not only enriching the repertoire of BE outcomes but also offering a more compact size and substantially reduced off‐target effects, thereby providing safer and more versatile tools for disease modeling and gene therapy. Together, these studies represent significant strides toward base editors with higher precision, broader applicability, and enhanced safety for clinical translation.

Beyond the nuclear genome, BE technology has been extended to mitochondrial DNA modification, with mitochondrial base editors enabling the precise construction of mitochondrial disease models [36, 37, 38] and providing valuable tools for both mechanistic investigations and therapeutic development. For instance, Zhang et al. report significant advancements in mitochondrial BE for the precise modeling of mitochondrial diseases [38]. They optimized mitochondrial base editors (mitoBEs) by engineering deaminases—specifically introducing the V28F mutation into TadA8e‐V106W for mitoABE and replacing APOBEC1 with CBE6d for mitoCBE, resulting in upgraded versions (mitoBEs v2) with substantially improved editing efficiency, strand specificity, and reduced off‐target effects on both the transcriptome and mitochondrial genome. Using circRNA‐encoded mitoBEs v2, the team achieved up to 82% editing efficiency in F0 mice at sites orthologous to human pathogenic mtDNA mutations, with no detectable nuclear genome off‐target effects. They successfully edited 68 out of 70 homologous mouse mtDNA sites and established multiple disease models, including mt‐Atp6 T8591C (Leigh syndrome model) and mt‐Nd5 A12784G (LHON model). These models recapitulated key disease phenotypes—bradycardia and vision loss, respectively. The study further demonstrated that mtDNA edits are tissue‐wide, durable, and maternally inheritable. Similarly, Chen et al. applied directed evolution to the deaminase TadA‐8e derived from CRISPR ABEs, obtaining highly active variants (e.g., A142R/L145W) [37]. By fusing these variants with a mitochondria‐targeted TALE system, they developed efficient and low‐off‐target ABEs for mitochondrial DNA (eTd‐mtABEs). This tool can efficiently introduce pathogenic point mutations in human cells and rats, successfully modeling mitochondrial diseases and demonstrating the great potential of BE for treating mitochondrial genetic disorders.

### 2.2 PE

PE, an advanced CRISPR‐based technology, enables the programmable replacement of targeted DNA sequences [39]. The system consists of a Cas9 nickase (Cas9n) fused to a reverse transcriptase, guided by a pegRNA that encodes both the target site and the desired edit [28]. The mechanism begins with the prime editor binding to its genomic target and creating a single‐strand nick [40]. The released 3′ DNA end then anneals to the pegRNA template, priming the reverse transcriptase to synthesize a new DNA strand that carries the edit. This edited 3′ strand subsequently displaces the original 5′ strand to install the modification, enabling a wide range of edits including substitutions, insertions, and deletions [41, 42, 43, 44, 45, 46] (Figure 3).

**FIGURE 3:**
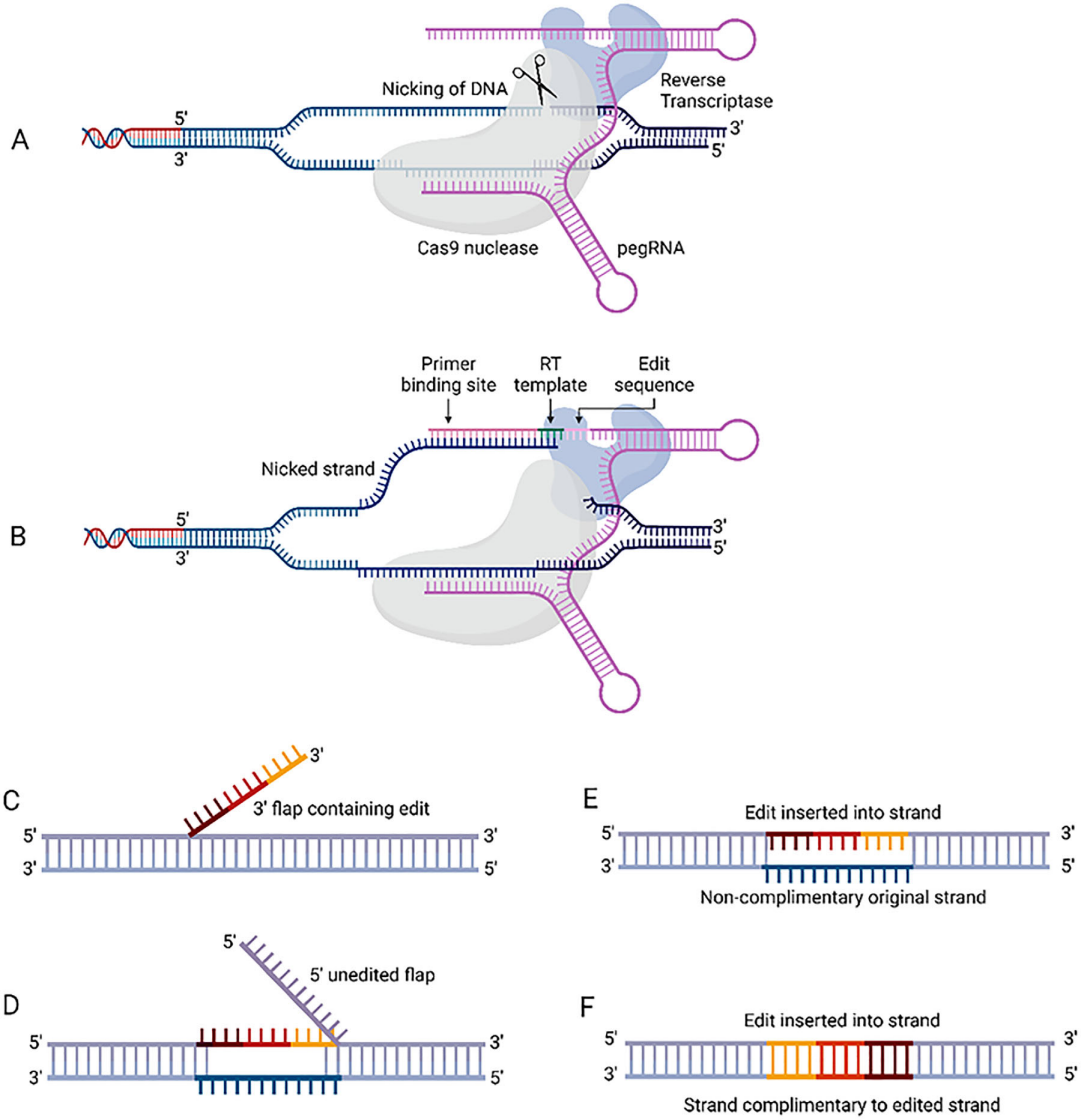
Molecular mechanism of prime editing. Prime editing is a precision genome editing technology that enables programmable insertions, deletions, and all 12 types of single‐base substitutions without DNA double‐strand breaks (DSBs) or exogenous donor templates. Panel A: Target site recognition and DNA nicking. The core prime editing complex consists of a Cas9 nickase (nCas9, RuvC‐inactivated, generates only single‐strand nicks) fused to a reverse transcriptase (RT), guided to the target locus by pegRNA. The pegRNA spacer first anneals to the complementary target DNA strand to anchor the complex; nCas9 then introduces a site‐specific nick on the non‐target strand, generating a free 3′ hydroxyl end that serves as the primer for subsequent reverse transcription. Panel B: Primer annealing and reverse transcription initiation. The multifunctional pegRNA contains three key 3′ extension elements beyond the Cas9‐binding spacer: a primer binding site (PBS) complementary to the nicked non‐target strand, an RT template, and the user‐defined edit sequence encoded within the template. After PBS annealing to the nicked DNA strand, RT uses the nicked 3′ end as a primer and the pegRNA RT template as a template to synthesize single‐stranded DNA carrying the desired edit. Panel C: Formation of the 3′ edited flap. Upon completion of reverse transcription, a single‐stranded 3′ flap containing the edit sequence and flanking wild‐type sequence is produced. The flanking genomic double‐stranded DNA remains intact throughout the process, reflecting the high locus specificity of the system. Panel D: 5′ unedited flap displacement and resolution. The 3′ edited flap hybridizes to the complementary target strand via sequence homology, displacing the original 5′ end of the nicked non‐target strand to form a 5′ unedited flap. Cellular flap endonuclease cleaves the unpaired 5′ flap, and the 3′ edited flap is ligated into the DNA backbone to form a heteroduplex DNA intermediate. Panels E and F: Heteroduplex resolution and permanent edit incorporation. The heteroduplex intermediate contains one edited strand and one unedited complementary strand. The cellular DNA mismatch repair (MMR) pathway is triggered by the heteroduplex and uses the edited strand as a template to repair the unedited strand. This permanently incorporates the desired edit into both strands of genomic DNA, completing the prime editing process.

To enhance this system, significant efforts have focused on improving efficiency through strategies such as inhibiting mismatch repair [47, 48], stabilizing pegRNAs [49, 50, 51], and engineering the reverse transcriptase [47, 52, 53, 54], leading to increasingly versatile and effective prime‐editing platforms. The unpredictability of editing outcomes, particularly the risk of propagating indel errors, poses a significant challenge for applications like multiplexed editing, gene drives, and gene therapy. Designing these systems requires a difficult balance between maximizing efficiency and minimizing errors, often necessitating extensive optimization of pegRNA and ngRNA design [55, 56, 57]. Chauhan et al. reported the development of high‐fidelity prime editors engineered for high precision and minimal genomic errors [58]. By introducing mutations in the Cas9 nickase domain that relax nick positioning and promote degradation of the competing 5′ strand, the researchers significantly reduced insertion and deletion (indel) errors during PE. They designed a series of optimized editors, including the very‐precise prime editor (vPE), which combines error‐suppressing mutations with a pegRNA‐stabilizing architecture (using the La protein). Compared to previous editors, vPE achieves comparable or higher editing efficiency while reducing indel errors by up to 60‐fold, resulting in edit‐to‐indel ratios as high as 543:1. This work demonstrates a novel strategy to enhance the fidelity of PE, enabling more accurate and reliable genome modifications.

### 2.3 Transposase System

Transposases are a class of sequence‐specific DNA‐binding enzymes that catalyze the excision and site‐specific integration of transposons—mobile genetic elements capable of moving between genomic loci. As core functional components of prokaryotic adaptive mobile genetic systems, transposases naturally enable efficient insertion of kilobase‐scale DNA sequences into target genomes, without requiring DNA DSBs or homologous donor templates. This addresses a critical limitation of canonical CRISPR‐Cas9 editing, which relies on low‐efficiency HDR for large transgene integration.

CRISPR‐associated transposases (CASTs) are naturally occurring bacterial systems that repurpose RNA‐guided, nuclease‐deficient CRISPR‐Cas machinery to direct the site‐specific insertion of kilobase‐scale transposons via Tn7‐like transposases [59, 60, 61]. These systems have evolved to couple the programmable RNA‐guided target recognition of CRISPR‐Cas with the robust, DSB‐free large‐fragment integration activity of transposases, eliminating the need for HDR and enabling efficient, targeted insertion of large genetic cargos. Tn7‐like CAST elements have co‐opted multiple CRISPR‐Cas subtypes, with Type I‐F and Type V‐K systems being the most well‐characterized to date. Type I‐F CASTs are particularly promising for therapeutic genome editing applications due to their high insertion efficiency, stringent on‐target specificity, predictable unidirectional insertion bias, high product purity, and low frequency of unwanted tandem insertions in bacterial models [62, 63, 64].

Despite this robust and specific activity in prokaryotic systems, naturally occurring Type I‐F CASTs exhibit minimal to no functional activity in human cells [65]. While the mechanistic basis for this loss of activity in mammalian cells is likely multifactorial, we and others have hypothesized that the native transposition catalysis or DNA binding steps of the bacterial system are inherently limited in eukaryotic chromatin environments—potentially as an evolutionary adaptation in bacteria to mitigate host fitness costs associated with excessive, off‐target transposition [66].

Substantial efforts have been made to engineer CAST systems to unlock their activity in human cells. Most notably, Witte et al. reported the development of evoCAST, an engineered and highly efficient CAST system for targeted, RNA‐guided insertion of large DNA sequences into the human genome. Using phage‐assisted continuous evolution (PACE), a high‐throughput directed evolution platform, the authors evolved the TnsB transposase subunit of a Type I‐F CAST system to dramatically improve its transposition and integration activity in human cells [67]. The optimized evoCAST system achieved integration efficiencies of 10%–25% for kilobase‐sized genetic cargos at multiple human genomic loci, representing a >420‐fold improvement over the wild‐type parental CAST system. Critically, evoCAST mediates targeted integration without inducing DSBs, minimizes the formation of unintended insertions and deletions (indels) and off‐target integration events, and maintains high product purity and unidirectional insertion specificity. The system was successfully applied to insert therapeutic transgenes, including the F9 cDNA for hemophilia B treatment and a CD19 CAR cassette for cancer immunotherapy, into clinically relevant genomic loci in human cells, demonstrating its strong potential for basic research and allele‐agnostic gene therapy applications.

### 2.4 Regulation of Gene Expression: CRISPRi and CRISPRa

CRISPR technology has evolved beyond genome editing to regulation of gene expression. Catalytically “dead” Cas9 (dCas9), through point mutations that retain DNA‐targeting ability but lack nuclease activity, enables regulation of gene expression. By directing dCas9 to gene promoters, transcription can be suppressed, an approach known as CRISPRi. In eukaryotic cells, dCas9 is often fused to transcriptional repressor domains such as KRAB [68, 69] to recruit chromatin‐modifying complexes and stably silence gene expression, offering an alternative to RNA interference. Conversely, CRISPRa employs dCas9 to recruit transcriptional activator domains [70, 71, 72] (e.g., VP64 and its derivatives) or to fuse with histone‐modifying enzymes [73, 74], such as demethylases or acetyl asesepigenetic editors (e.g., the histone acetyltransferase p300) to upregulate target genes.

### 2.5 Expanded CRISPR Toolkits Based on Cas12 and Cas13 Systems

Beyond the canonical Cas9, BE, and PE platforms detailed above, Class 2 Type V (Cas12) and Type VI (Cas13) CRISPR‐Cas systems have emerged as a versatile, expandable set of tools for genome/transcriptome engineering and molecular diagnostics, owing to their unique target substrate preferences and specialized biochemical properties.

#### 2.5.1 Molecular Mechanisms of Cas12

Cas12 effectors are the signature single‐subunit RNA‐guided endonucleases of Class 2 Type V CRISPR‐Cas systems, with Cas12a (formerly Cpf1) being the most extensively characterized ortholog, alongside other functionally diverse subtypes including Cas12b, Cas12c, and Cas12d [75]. Unlike the well‐characterized Cas9 system, Cas12 effectors exhibit two intrinsic catalytic activities: a metal‐dependent DNase activity harbored in a single conserved RuvC nuclease domain, and an autonomous pre‐CRISPR RNA (pre‐crRNA) processing activity that eliminates the requirement for trans‐activating crRNA (tracrRNA) or auxiliary RNase III [75]. This self‐sufficient crRNA maturation capability enables Cas12 to efficiently process multi‐spacer crRNA arrays, making it a robust tool for multiplexed genome editing [76]. The RuvC domain, which contains a conserved DED catalytic triad coordinated with divalent cations (typically Mg^2^^+^), is the sole catalytic center responsible for all DNA cleavage events, distinguishing Cas12 from Cas9 that uses separate HNH and RuvC domains to cleave target and non‐target DNA strands, respectively.

Target recognition by Cas12 is initiated by the identification of a 5' T‐rich PAM, most commonly a TTTV consensus sequence (V = A, C, G) located upstream of the target protospacer on the non‐target DNA strand. PAM recognition is mediated by the PAM‐interacting (PI) domain and the tryptophan‐aspartic acid (WED) domain of Cas12, which triggers local unwinding of the double‐stranded DNA (dsDNA) duplex and initiates base pairing between the 3' variable spacer of the mature crRNA and the complementary target DNA strand, driving the formation of an R‐loop structure. Full R‐loop stabilization induces a dramatic conformational rearrangement of the Cas12 protein, which unlocks the catalytic potential of the RuvC domain. For dsDNA substrates, the single RuvC active site executes sequential, staggered cleavage of both DNA strands: it first nicks the non‐target strand, followed by cleavage of the target strand, generating 4–5 nt 5' overhangs distal to the PAM site, outside the seed region of the crRNA‐DNA heteroduplex.

A defining functional hallmark of Cas12 is its target‐activated non‐specific collateral cleavage activity. Upon recognition and binding of a fully complementary dsDNA target, the activated RuvC domain unleashes robust, promiscuous degradation of any nearby ssDNA molecules in trans, a property that persists as long as Cas12 remains bound to its target DNA. This collateral DNase activity has been widely harnessed for the development of ultrasensitive nucleic acid detection platforms, including the DNA endonuclease‐targeted CRISPR trans reporter (DETECTR) system. Catalytically dead Cas12 (dCas12) variants, generated by inactivating mutations in the RuvC domain, retain sequence‐specific DNA binding capacity, enabling their repurposing for targeted epigenome editing, transcriptional modulation, and live‐cell genomic imaging without inducing permanent DSBs [75].

#### 2.5.2 Molecular Mechanisms of Cas13

Cas13 is the sole effector protein of Class 2 Type VI CRISPR‐Cas systems, the only known class 2 CRISPR effectors that exclusively target RNA rather than DNA, with 11 functionally validated subtypes identified to date, among which Cas13a, Cas13b, and Cas13d are the most widely utilized in mammalian and prokaryotic systems [77]. All Cas13 orthologs are single‐subunit RNA‐guided ribonucleases (RNases) defined by two conserved higher eukaryotes and prokaryotes nucleotide‐binding (HEPN) domains, each containing a characteristic R‐X‐H catalytic motif that is essential for RNase activity. Structurally, Cas13 is organized into two main lobes: a recognition (REC) lobe responsible for crRNA binding and target RNA recognition, and a nuclease (NUC) lobe that houses the two HEPN domains and mediates catalytic activation [77].

Similar to Cas12, Cas13 possesses intrinsic, metal‐independent RNase activity that enables autonomous processing of pre‐crRNA transcripts into mature guide RNAs, without the need for tracrRNA or additional host factors. This processing activity occurs within the conserved direct repeat (DR) region of the pre‐crRNA, generating mature crRNAs with a conserved 3' DR stem‐loop structure and a 20–30 nt 5' spacer sequence that mediates complementary target RNA binding. Unlike DNA‐targeting CRISPR systems, Cas13 does not require a PAM sequence; instead, target recognition is governed by sequence complementarity between the crRNA spacer and the single‐stranded RNA (ssRNA) protospacer, as well as the presence of a protospacer flanking site (PFS), a short sequence motif located at the 3' end of the target protospacer [78].

Upon binding to a fully complementary ssRNA target, the Cas13‐crRNA complex undergoes a substantial conformational rearrangement, which brings the two spatially separated HEPN domains into close proximity to form a single, catalytically active composite RNase pocket [77]. This activation triggers two distinct catalytic activities: first, site‐specific cis‐cleavage of the complementary target RNA, typically occurring at uracil‐rich regions within the protospacer; second, a robust, non‐specific trans‐collateral RNase activity, the most prominent functional feature of Cas13. Once activated, Cas13 promiscuously degrades any nearby ssRNA molecules regardless of their sequence complementarity to the crRNA, a property that is constitutively active as long as the enzyme remains in its target‐bound conformation [79]. This robust collateral activity has established Cas13 as the foundational component of the Specific High‐Sensitivity Enzymatic Reporter UnLOCKing (SHERLOCK) platform for ultrasensitive RNA detection, including viral pathogen diagnostics.

Catalytically dead Cas13 (dCas13) variants, generated by inactivating mutations in the R‐X‐H motifs of the HEPN domains, lose all RNase activity but retain full sequence‐specific RNA binding capacity. This has enabled the development of a broad suite of RNA‐targeting tools, including targeted transcript knockdown, alternative splicing modulation, site‐specific epitranscriptomic editing, and live‐cell RNA imaging, all of which operate without altering the underlying genomic DNA sequence, offering a reversible and tunable approach for gene expression regulation [78].

#### 2.5.3 Optimization and Innovation of Cas12 and Cas13 Systems

Substantial efforts have been made in recent years to expand the Cas12/Cas13 editing toolkit, with a particular focus on compact effectors to overcome delivery bottlenecks for in vivo therapeutic applications.

Yoon et al. used a structure‐guided bioinformatics pipeline to identify Cas13an, an effector from an ancestral Cas13 clade that is approximately one‐third the size of well‐characterized canonical Cas13 effectors (e.g., Cas13d) [80]. Cas13an retains robust RNA‐guided RNA cleavage activity and mediates broad‐spectrum defense against diverse bacteriophages, laying a foundation for microbial phage resistance engineering. Notably, unlike larger Cas13 subtypes that rely on two separate Higher Eukaryotes and Prokaryotes Nucleotide‐binding (HEPN) domain active sites, Cas13an uses a single HEPN domain active site to mediate both CRISPR RNA (crRNA) processing and target RNA cleavage. This dual functionality is hypothesized to reflect the ancestral evolutionary state of Cas13 effectors, with phylogenetic analyses further supporting that modern Cas13 systems evolved from the fusion of two defense‐associated HEPN ribonucleases.

In parallel, Zheng et al. [81] characterized the compact Type V CRISPR‐Cas effector Cas12h1 in 2025, which functions primarily as a site‐specific nickase, catalyzing single‐strand cleavage of the non‐target DNA strand without inducing DSBs. A key advantage of Cas12h1 is its recognition of a relaxed, broad PAM sequence (5′ ‐DHR‐3′), which greatly expands its targetable genomic range relative to Cas9 and other Cas12 variants with strict PAM requirements. Furthermore, rational protein engineering yielded a high‐fidelity variant, Cas12h1h, which exhibits enhanced discrimination against single‐base mismatches between the guide RNA and off‐target genomic loci, reducing unintended off‐target editing events and improving its suitability for therapeutic genome editing applications.

Beyond their utility in genome and transcriptome engineering, the non‐specific collateral cleavage activity of Cas12 and Cas13 effectors has enabled their extensive development as next‐generation nucleic acid diagnostic platforms, with direct clinical translation potential. For these diagnostic systems, Cas13 or Cas12 nucleases are programmed via a guide RNA to bind a user‐defined target nucleic acid (RNA for Cas13, DNA for Cas12); target binding triggers robust non‐specific collateral cleavage of nearby reporter molecules, generating a quantifiable, detectable fluorescent signal for highly specific and sensitive nucleic acid detection [82, 83, 84]. A representative clinical application of this technology is DISCoVER (Diagnostics with Coronavirus Enzymatic Reporting), a rapid, extraction‐free nucleic acid test developed for SARS‐CoV‐2 detection in human saliva samples which integrates direct chemical lysis of clinical samples, multiplexed loop‐mediated isothermal amplification (LAMP) coupled with T7 RNA polymerase‐mediated transcription, and Cas13‐mediated fluorescent reporter detection in a single streamlined workflow, making it compatible with low‐resource and point‐of‐care clinical settings [85].

## 3 Clinical Translation

Over the past 3 years, CRISPR technology has transitioned from a promising research tool to a clinically validated therapeutic modality, with multiple candidates advancing through late‐stage trials and receiving regulatory approval. Each therapy employs a unique combination of editing platform and delivery system, tailored to the specific pathophysiology of the target disease. To provide a comprehensive and up‐to‐date reference for clinicians and researchers, we summarize the core characteristics, clinical development status, and key efficacy data of 11 leading CRISPR‐based therapeutic candidates in Table 1.

**TABLE 1:** Clinical translation landscape of leading CRISPR‐based therapeutic candidates.

Representative therapies	Delivery system	Lead indications	Mechanism of action (MOA)	Clinical stage	Key clinical results (latest follow‐up)
Exagamglogene autotemcel (exa‐cel) (NCT03745287, NCT03655678)	Ex vivo Cas9 RNP electroporation	Sickle cell disease (SCD), transfusion‐dependent β‐thalassemia (TDT)	CRISPR‐Cas9 disruption of BCL11A erythroid enhancer to reactivate fetal hemoglobin (HbF)	Completed Approved (UK/US/EU)	97% of SCD patients (*n* = 29) were free of severe vaso‐occlusive crises for 1 year or more [86] 91% of TDT patients (*n* = 32) achieved transfusion independence [87]
VERVE‐101 (NCT05398029)	Systemic lipid nanoparticles (LNPs)	Heterozygous familial hypercholesterolemia (heFH), atherosclerotic cardiovascular disease	Adenine base editor (ABE)‐mediated permanent hepatic PCSK9 inactivation	Terminated at Phase Ib	Terminated after a serious drug‐related adverse event, with elevated ALT and thrombocytopenia [88]
VERVE‐102 (NCT06164730)	GalNAc‐conjugated LNPs	Heterozygous familial hypercholesterolemia (heFH); premature coronary artery disease	ABE‐mediated hepatic PCSK9 inactivation	Phase 1	A single infusion of VERVE‐102 resulted in dose‐dependent, substantial, and sustained reductions in PCSK9 and LDL cholesterol levels (*n* = 35) [89]
VERVE‐201 (NCT06451770)	GalNAc‐conjugated LNPs	Homozygous familial hypercholesterolemia (hoFH)	ABE‐mediated permanent hepatic ANGPTL3 inactivation	Phase I (Recruiting)	Preclinical mouse and NHP data [90]
BEAM‐101 (NCT05456880)	Ex vivo ABE RNP electroporation	Sickle cell disease and severe vaso‐occlusive crises	ABE introduces A→G substitutions in HBG1/2 promoters to disrupt BCL11A binding and reactivate HbF [91]	Phase I/II (active)	Near‐complete HbF reactivation (≥ 98% of RBCs); near‐elimination of HbS‐only RBCs (< 2%); normalized erythroid rheology [92]
k‐abe (N‐of‐1 compassionate use)	Systemic LNPs	Neonatal‐onset carbamoyl phosphate synthetase 1 (CPS1) deficiency	Customized ABE corrects pathogenic nonsense variant (Q335X) in hepatic CPS1 gene	Compassionate use (completed)	Increased dietary protein tolerance; no hyperammonemic crises during 8‐month follow‐up [93]
PM359 (NCT06559176)	Ex vivo prime editor mRNA electroporation	p47phox‐deficient chronic granulomatous disease	Prime editing corrects 2‐nucleotide deletion in NCF1 gene in autologous CD34+ HSPCs	Phase I/II (Enrolling by invitation)	69% and 83% restoration of neutrophil NADPH oxidase function (*n* = 2); no serious treatment‐related adverse events; no detectable off‐target edits [94]
ESO‐T01 (NCT06691685)	Engineered lentiviral vector with VSV‐G mutation, anti‐TCR nanobody for T‐cell targeting, CD47 overexpression and MHC‐I knockout for immune evasion	Relapsed/refractory multiple myeloma	Delivers anti‐BCMA CAR transgene in vivo to reprogram endogenous T cells into functional CAR‐T cells in situ; the expanded CAR‐T cells specifically recognize and kill BCMA‐expressing myeloma cells	Phase I (active)	Two stringent complete responses, 2 partial responses; all patients achieved bone marrow MRD negativity by day 28; active against extramedullary disease [95]
INT2104 (NCT06539338)	Engineered lentiviral vector platform: (1) Gen 2.1 detargeted fusogen (mutated VSV‐G to eliminate non‐specific binding) (2) Membrane‐anchored CD7‐targeting scFv Route: single intravenous infusion; no ex vivo cell manufacturing, no lymphodepleting conditioning chemotherapy required	Relapsed/refractory B‐cell malignancies (B‐cell non‐Hodgkin lymphoma, precursor lymphoblastic leukemia/lymphoma, Burkitt lymphoma)	Following a single intravenous infusion, the CD7‐targeted lentiviral vector selectively transduces endogenous CD7^+^ T and NK cells in vivo, prompting them to express a fully human anti‐CD20 CAR (4‐1BB/CD3ζ signaling domains). The de novo generated functional CAR‐T and CAR‐NK cells specifically eliminate CD20^+^ malignant B cells, with no need for ex vivo cell manufacturing or lymphodepleting preconditioning	Phase I Multicenter, open‐label, two‐part design (dose escalation + dose expansion) recruitment complete	Preclinical: Selective CD7‐targeted transduction of T/NK cells with dose‐dependent CD20^+^ tumor cytotoxicity in vitro. Single infusion achieves rapid B‐cell depletion, complete tumor regression and prolonged survival in humanized mice. Durable B‐cell depletion (> 1 year) without lymphodepletion in cynomolgus NHPs; GLP toxicology shows favorable safety with no CRS or neurotoxicity [96]

*Note*: N‐of‐1 therapy: k‐abe was developed and administered to a single patient under compassionate use protocol, representing the first successful personalized in vivo CRISPR‐base editing therapy.

Abbreviations: AAV, adeno‐associated virus; ABE, adenine base editor; GalNAc, N‐acetylgalactosamine; HbF, fetal hemoglobin; HSPC, hematopoietic stem and progenitor cell; LNP, lipid nanoparticle; MOA, mechanism of action; NHP, non‐human primate; RNP, ribonucleoprotein; sCR, stringent complete response; VOC, vaso‐occlusive crisis.

### 3.1 β‐Thalassemia and SCD

#### 3.1.1 Approved Therapy: Exagamglogene Autotemcel (exa‐cel)

The most vanguard success story is the development of ex vivo CRISPR‐Cas9 gene‐edited cell therapies for hemoglobinopathies, specifically SCD and β‐thalassemia caused by mutations in the β‐globin gene (HBB). The pioneering strategy, exemplified by therapies such as exagamglogene autotemcel (exa‐cel, marketed as Casgevy), does not directly correct the disease‐causing mutation in the HBB gene. Instead, it employs a sophisticated “gene disruption” approach to target the BCL11A gene, a key transcriptional repressor of fetal hemoglobin (HbF) [97]. Hematopoietic stem and progenitor cells (HSPCs) are harvested from the patient and edited ex vivo using CRISPR‐Cas9 to disrupt the BCL11A erythroid‐specific enhancer. This disruption reactulates the production of HbF, which is unaffected by the HBB mutations. The therapeutic editing is achieved by electroporating CD34^+^ HSPCs with Cas9 ribonucleoprotein (RNP) to disrupt an erythroid‐specific enhancer within the BCL11A gene. Following myeloablative conditioning and reinfusion of these edited HSPCs into the patient, the edited cells produce red blood cells (RBCs) containing high levels of HbF. Elevated HbF compensates for the defective adult hemoglobin, effectively ameliorating the pathophysiology of the diseases [98]. Clinical trials for exa‐cel (NCT03745287, NCT03655678) have demonstrated remarkable and sustained efficacy. In the phase I/II clinical trial, the first clinical results of exa‐cel in two index patients with transfusion‐dependent β‐thalassemia (NCT03655678) and severe SCD were reported in 2021 [97]. Autologous CD34^+^ cells were edited ex vivo via Cas9 RNP electroporation to disrupt the BCL11A erythroid enhancer. Over 12 months of follow‐up, both patients exhibited sustained high‐level HbF induction and achieved curative clinical benefits with acceptable safety. Then the pivotal phase 3 CLIMB SCD‐121 trial (NCT03745287) established the curative potential of exa‐cel for severe SCD. This open‐label, single‐arm study enrolled 44 patients who received autologous CD34^+^ cells edited via CRISPR‐Cas9 to disrupt the BCL11A erythroid enhancer, followed by busulfan myeloablation. With a median follow‐up of 19.3 months, 29 of 30 evaluable patients (97%) achieved the primary endpoint of ≥ 12 consecutive months free from severe VOCs, and all 30 patients (100%) avoided VOC‐related hospitalizations [86]. Exa‐cel induced durable high‐level allelic editing (≥ 70% in peripheral blood, 86.1% in bone marrow CD34^+^ cells at month 6), sustained HbF elevation, and resolution of chronic hemolysis [86]. Adverse events were predominantly attributable to conditioning, with no exa‐cel‐related serious toxicities, graft failure, or secondary malignancies reported [86]. Meanwhile, the pivotal phase 3 CLIMB THAL‐111 trial established the curative potential of exa‐cel for transfusion‐dependent β‐thalassemia. This open‐label, single‐arm study enrolled 52 patients who received autologous CD34^+^ cells edited via CRISPR‐Cas9 to disrupt the BCL11A erythroid enhancer, followed by pharmacokinetically adjusted busulfan conditioning. With a median follow‐up of 20.4 months, 32 of 35 evaluable patients (91%) achieved the primary endpoint of ≥ 12 consecutive months of transfusion independence with normal hemoglobin levels [87]. Exa‐cel induced sustained pancellular HbF expression (mean 11.9 g/dL at month 6) and stable allelic editing (≥64% in peripheral blood, 78% in bone marrow CD34^+^ cells) [87]. Adverse events were predominantly attributable to conditioning, with only two cases of exa‐cel‐related serious adverse events that fully resolved, and no cases of graft failure or secondary malignancies reported [87]. Based on these groundbreaking results, exa‐cel received landmark regulatory approvals from the UK MHRA, the US FDA, and the European Commission in late 2023 and early 2024 [99, 100].

Patient‐reported outcomes from 42 evaluable patients (30 adults, 12 adolescents) with severe SCD who received exa‐cel (median follow‐up 33.6 months) demonstrated broad and durable health‐related quality of life (HRQoL) benefits. By month 6, adults achieved clinically significant improvements in general health status (EQ‐5D‐5L), transplant‐related well‐being (FACT‐BMT), and SCD‐specific outcomes (ASCQ‐Me), including marked reductions in pain frequency and severity. Adolescents showed comparable improvements in pediatric HRQoL measures (PedsQL) and pain scores, with benefits sustained through 24 months [101].

Similarly, exa‐cel conferred broad, durable, clinically meaningful HRQoL improvements in transfusion‐dependent β‐thalassemia patients in the CLIMB THAL‐111/131 trials. Among 54 evaluable patients (35 adults, 19 adolescents) with a median follow‐up of 38.4 months, adults showed sustained improvements in general health (EQ‐5D‐5L) and transplant‐related well‐being (FACT‐BMT) through 48 months. Adolescents had significant gains in PedsQL scores across physical and psychosocial domains, with benefits emerging as early as 6–9 months and eliminating transfusion‐related HRQoL fluctuations [102].

#### 3.1.2 Active Beam‐101

Beyond Cas9 nuclease‐based therapies, BE has emerged as a safer and more precise alternative for hemoglobinopathy treatment. Several studies demonstrate the therapeutic potential of adenine BE in HSPCs for treating haemoglobinopathies [92, 103, 104]. One strategy directly corrects the causative mutation. By engineering a custom ABE (ABE8e‐NRCH) to directly convert the pathogenic *HBB* ^S^ allele into the non‐pathogenic *HBB* ^G^ (Makassar β‐globin) variant in human HSPCs without introducing DSBs, ex vivo delivery of ABE8e‐NRCH mRNA and sgRNA into patient‐derived HSPCs achieved up to 80% on‐target editing with minimal indels or bystander edits [104]. Transplantation of edited cells into mouse models led to durable correction, restored normal blood parameters, and significantly reduced disease symptoms. Unlike Cas9 nuclease, BE avoided p53 activation and large DNA deletions.

An alternative, mutation‐agnostic approach targets HbF reactivation because elevated HbF can compensate for the defective adult hemoglobin, and effectively ameliorate the pathophysiology of the diseases. Xu et al. developed an antibody‐free LNP platform (LNP‐168) to deliver ABE8e mRNA and an optimized sgRNA (sgRNA‐25) targeting the *HBG1/2* promoter in vivo [103]. By optimizing ionizable lipids, they developed LNP‐168, which enables efficient editing of the HBG1/2 promoter in HSCs to reactivate HbF. In humanized mice engrafted with β‐thalassemia patient HSCs, intravenous LNP‐168‐ABE8e‐HBG achieved clinically relevant editing, restored globin balance, and improved erythroid maturation without the need for HSC mobilization, harvesting, or chemotherapy conditioning. Incorporating miR‐122 target sequences reduced liver off‐target editing while preserving bone marrow activity.

This HbF‐reactivation strategy has now advanced to clinical translation. The phase 1/2 open‐label BEACON trial (NCT05456880) interim data evaluated BEAM‐101, an ex vivo adenine base‐edited autologous CD34^+^ HSPC therapy introducing A→G substitutions in the HBG1/HBG2 promoters, in three patients with severe SCD and recurrent vaso‐occlusive crises (VOCs) [92]. This edit disrupts BCL11A binding, thereby reactivating HbF expression in patients with SCD and severe VOCs. BEAM‐101 achieved rapid, near‐complete HbF expression, near‐elimination of HbS‐only RBCs, normalized adhesion and hemorheology, and reversal of sickling phenotype [92].

Treatment induced near‐complete remodeling of RBC hemoglobin profiles: total HbF‐positive RBCs increased from 20.8% to 48.5% at baseline to > 99% by Month 2, while RBCs expressing exclusively sickle hemoglobin (HbS) were nearly eliminated (< 0.4% at Month 2). All patients achieved HbF levels > 19 pg per F‐cell, exceeding the 10 pg protective threshold for sickling. Vascular adhesion (VCAM1 and P‐selectin mediated) dropped significantly below established VOC risk thresholds by Month 3. Treated RBCs showed sickling kinetics comparable to sickle cell trait (SCT) samples, with normalized hemorheological parameters and complete resolution of abnormal sickle cell morphology by Month 4–6. These data support the transformative therapeutic potential of HBG1/HBG2 promoter BE for SCD [92].

#### 3.1.3 PE Demonstrates Therapeutic Potential in SCD Preclinical Animal Models

In addition, two recent studies have demonstrated the potential of PE to correct the SCD mutation directly in hematopoietic stem cells (HSCs), using complementary ex vivo and in vivo approaches. Everette et al. employed an optimized PE3max system delivered via RNA electroporation to correct the HBB^5^ allele in patient‐derived HSPCs ex vivo, achieving 15%–41% editing with minimal indels [105]. Following transplantation into immunodeficient mice, edited cells engrafted efficiently and maintained therapeutic editing for 17 weeks, producing HbA‐expressing erythrocytes resistant to hypoxia‐induced sickling. In parallel, Li et al. developed an in vivo approach using a helper‐dependent adenoviral vector (HDAd5/35++) to deliver PE components directly to HSCs in a humanized SCD mouse model [106]. A single intravenous injection following HSC mobilization, combined with low‐dose drug selection, resulted in ∼40% correction of β^5^ alleles and replacement of 43% of HbS with HbA, leading to substantial phenotypic amelioration including normalized hematological parameters and reduced organ pathology. Both studies reported minimal off‐target editing and confirmed that long‐term repopulating HSCs were successfully edited. Together, these findings establish PE as a precise and promising strategy for SCD gene therapy, offering the potential for both ex vivo and in vivo approaches that avoid DSBs and exogenous DNA templates [106].

### 3.2 Ex Vivo CAR‐T Cell Therapy

#### 3.2.1 Engineering Strategies and Clinical Translation

Lu et al. [107] report results from a first‐in‐human phase I clinical trial of CRISPR‐Cas9 PD‐1‐edited T cells in patients with advanced non‐small‐cell lung cancer (ClinicalTrials.gov NCT02793856). Another pioneering CRISPR‐Cas9 clinical trial ex vivo edit autologous T cells by knocking out endogenous T cell receptor (TCR) and the immune checkpoint PD1, and introducing a tumor‐antigen‐specific TCR before re‐infusing the cells back into patients [108]. Autologous CAR‐T cell products using retroviral or lentiviral vectors to engineer T cells to target cancer cell markers like CD19 or B‐cell maturation antigen (BCMA) in B‐cell malignancies and multiple myeloma have been proved [109]. Universal CAR‐T cells are engineered using allogenic T cells by eliminating immune incompatibility. The main strategy to build universal CAR‐T cells with CRISPR/Cas9 includes the simultaneous knockout of several key genes: the TCR genes, β‐2 microglobulin (B2M), and immune checkpoint genes such as PD1. Disruption of TCR genes (TRAC and TRBC) eliminates the endogenous TCR, thereby preventing graft‐versus‐host disease (GVHD) by stopping recognition of host tissues as foreign. Deleting B2M, a component of HLA class I molecules, prevents recognition of the CAR‐T cells by the patient's immune system as foreign cells, thus reducing graft rejection. Additionally, simultaneous knockout of PD1 enhances antitumor activity by preventing inhibitory signaling that leads to T‐cell exhaustion [110]. Mikkelsen et al. [111] knock out B2M and REGNASE‐1 without DSB using *Staphylococcus aureus* Cas9 (SaCas9) mRNA base editors and insert the anti‐CD19 CAR gene into the TCR α constant (TRAC) locus using *Streptococcus pyogenes* (SpCas9) with minimal translocations. Together, these modifications are designed to create safer, more potent, and persistent allogeneic CAR‐T cells.

#### 3.2.2 Long‐Term Efficacy and Safety of JULIET, ZUMA‐1, and TRANSCEND

Long‐term efficacy and safety of CAR‐T cell therapies are key to assessing the therapeutic potential of this treatment. We compare efficacy and safety of CD19‐directed CAR‐T cell therapies in patients with relapsed/refractory (r/r) diffuse B‐cell lymphomas (DLBCL) from the latest follow‐up of JULIET [112], ZUMA‐1 [113], and TRANSCEND trials [114] (Table 2). Maziarz et al. [112] report a 5‐year analysis of the JULIET trial of tisagenlecleucel in patients with r/r DLBCL with a median follow‐up duration of 74.3 months (ClinicalTrials.gov:NCT02445248). Note that 5‐year relapse‐free probability was 61% among responders. Overall survival (OS) probability at 5 years was 32% for all the patients and 56% for those achieving complete or partial response. This provides the strongest evidence for the long‐term durability of tisagenlecleucel. Neelapu et al. [113] report a 5‐year follow‐up of ZUMA‐1, which supports the curative potential of axicabtagene ciloleucel in r/r DLBCL (NCT02348216). In this analysis with a median follow‐up of 63.1 months, axicabtagene ciloleucel demonstrated a remarkable 31% of patients maintaining ongoing responses at 5 years. The estimated 5‐year overall survival (OS) rate was 42.6%, with a median OS of 25.8 months. Importantly, the study provided new insights into the mechanism of long‐term remission, showing that protracted B‐cell aplasia was not a prerequisite for durable responses, as polyclonal B‐cell recovery was observed in 91% of evaluable patients by 3 years. No new long‐term safety concerns emerged with extended follow‐up, further solidifying its favorable risk‐benefit profile. Similarly, the TRANSCEND NHL 001 trial investigating lisocabtagene maraleucel (liso‐cel) has reported promising 2‐year follow‐up results [114] (NCT02631044 and NCT03435796). With a median follow‐up of 19.9 months, liso‐cel achieved an objective response rate (ORR) of 73% and a complete response (CR) rate of 53% in efficacy‐evaluable patients (*N* = 257) with heavily pre‐treated R/R LBCL (median of 3 prior lines). The median OS was 27.3 months, with an estimated 2‐year OS rate of 50.5%. The durability of response is supported by a median duration of response (DOR) of 23.1 months and a 2‐year DOR rate of 49.5%. Regarding safety, liso‐cel maintained a manageable profile, with low rates of grade ≥ 3 cytokine release syndrome (CRS) (2%) and neurological events (10%) within the 90‐day treatment‐emergent period. The most common grade ≥ 3 adverse events were hematologic, including neutropenia (60%) and anemia (37%), primarily during the treatment‐emergent period. Importantly, no new safety signals emerged during extended follow‐up, further supporting its long‐term safety. These data demonstrate that liso‐cel induces durable remissions in a significant proportion of patients with high‐risk, multi‐refractory disease.

**TABLE 2:** Comparative table: long‐term follow‐up of CD19 CAR‐T therapies in R/R LBCL.

	Aspect	Axicabtagene ciloleucel (Axi‐cel) ZUMA‐1 [113]	Lisocabtagene maraleucel (Liso‐cel) transcend NHL 001 [114]	Tisagenlecleucel (Tisa‐cel) JULIET [112]
Clinical trial	NCT number	NCT02348216	NCT02631044	NCT02445248
	Study follow‐up	63.1 months	19.9 months	74.3 months
	Number of infused patients	101	270	115
Product characteristics	Trade name	Yescarta (Kite)	Breyanzi (Bristol Myers Squibb)	Kymriah (Novartis)
	CAR structure	CD19 scFv, CD28 co‐stimulatory domain	CD19 scFv, 4‐1BB co‐stimulatory domain	CD19 scFv, 4‐1BB co‐stimulatory domain
	Product design	Unfractionated CD4^+^/CD8^+^ T cell mixture	Separate CD4^+^ and CD8^+^ CAR‐T cell products infused sequentially at a fixed 1:1 ratio	Unfractionated CD4^+^/CD8^+^ T‐cell mixture
	Recommended dose	2 × 10^6^ CAR‐T cells/kg	100 × 10^6^ CAR^+^ T cells	0.6–6.0 × 10^8^ CAR‐T cells
	Administration	Single infusion	Two sequential infusions (CD8^+^ then CD4^+^)	Single infusion
Efficacy	ORR	83%	73%	53%
	CR Rate	58%	53%	39%
	Median OS	25.8 months	27.3 months	11.1 months
	Long‐term OS rate	5‐year OS: 42.6%	2‐year OS: 50.5%	5‐year OS: 31.7%
	OS in CR/responders	5‐year OS: 64.4% (in CR)	Median OS: 48.5 months (in CR)	5‐year OS: 55.8% (in CR/PR)
	Durability (median DOR)	11.1 months	23.1 months	Not reached
Safety	≥ 3 CRS	11%	2%	23%^a^
	≥ 3 Neurotoxicity	30%	10%	11%^a^
	Secondary malignancies	1 (1%) (not treatment‐related)	22 (8.1%) total: five (2%) in treatment period, 17 (7%) post‐treatment Most common: nonmelanoma skin cancer (nine patients)	15 events in 13 patients (11.3%) No secondary T‐cell malignancies reported

*Note*: This table summarizes the key efficacy, safety, product design characteristics, and long‐term follow‐up data of three FDA‐approved CD19 CAR‐T therapies for the treatment of R/R LBCL. All clinical data are derived from the long‐term follow‐up results of three landmark multicenter clinical trials: ZUMA‐1 (axicabtagene ciloleucel, axi‐cel) [113], TRANSCEND NHL 001 (lisocabtagene maraleucel, liso‐cel) [114], and JULIET (tisagenlecleucel, tisa‐cel) [112]. Grade ≥ 3 adverse event rates are reported from the primary analysis of the corresponding clinical trial.

Abbreviations: CD, cluster of differentiation; CR, complete response; CRS, cytokine release syndrome; DOR, duration of response; ORR, overall response rate; OS, overall survival; scFv, single‐chain variable fragment.

^a^ Important Note on Safety Comparison: The JULIET trial (Tisa‐cel) used the Penn grading scale for CRS, which is generally more stringent and leads to higher rates of severe CRS compared to the Lee criteria used in the TRANSCEND (Liso‐cel) and ZUMA‐1 (Axi‐cel) trials. Therefore, the incidences of ≥ 3 CRS are not directly comparable.

### 3.3 In Vivo CAR‐T Cell Therapy

Despite significant clinical successes, the broader application of autologous and allogeneic CAR‐T cell therapies into wider indications and patient populations is limited due to complex and lengthy ex vivo manufacturing processes, limited production capacity, and the necessity for chemotherapy‐based lymphodepletion conditioning [115].

In vivo CAR‐T cell therapy is emerging as a transformative alternative designed to circumvent these challenges, offering a potentially more scalable “off‐the‐shelf” approach that eliminates the need for ex vivo cell manipulation and lymphodepletion. The core mechanism of this strategy involves the direct, in situ genetic engineering of a patient's own immune cells, such as T cells, natural killer (NK) cells, or macrophages by the systemic administration of viral vectors or lipid nanoparticle (LNP) formulations carrying CAR‐encoding payloads. Viral vectors enable permanent CAR integration, leading to the generation of engineered cells capable of long‐term persistence and expansion. In contrast, non‐viral platforms like LNP‐delivered mRNA produce transient CAR expression, which inherently limits the duration of activity but may offer enhanced safety profiles.

Early proof‐of‐concept studies established two principal strategies for in vivo CAR‐T cell generation. Smith et al. [116] first reported the use of DNA‐carrying nanoparticles to introduce CAR genes into T cells, achieving long‐term leukemia remission. Similarly, Pfeiffer et al. [117] demonstrated that CD8^+^ T cells could be directly engineered in vivo using CD8‐targeted lentiviral vectors to generate functional human CD19 CAR‐T cells.

#### 3.3.1 Viral Vectors

Engineered lentiviral or gamma‐retroviral vectors are used to deliver genomically integrate CAR transgenes in a cell‐selective manner. A pivotal challenge lies in achieving highly specific gene transfer into patients’ T cells, which necessitates preventing vector uptake by non‐target cells. Such precision not only enhances safety by avoiding off‐target effects but also improves transduction efficiency by minimizing the nonspecific sequestration of vector particles. To address this, the viral envelope proteins used to pseudotype lentiviral vectors have been systematically redesigned by abolishing interactions with native cellular receptors to reduce off‐target binding and displaying high‐affinity targeting moieties that recognize T cell–specific surface markers [118, 119].

Nicolai et al. present the first preclinical evidence that in vivo CAR T‑cell therapy is efficacious in non‑human primates (NHPs) using the VivoVec lentiviral platform [120]. By engineering the surface of vector particles (VVPs) to display a multidomain fusion protein that provides integrated T‑cell activation and costimulatory signals, enhanced transduction and robust generation of anti‑CD20 CAR T cells in NHPs and the complete B‑cell depletion for more than 10 weeks have been reported.

Furthermore, as the first clinical candidate from a lentiviral platform, INT2104 embodies the core design of a lentiviral vector for in vivo targeted delivery [96]. It utilizes a detargeted viral fusogen (Gen 2.1 Fusogen) to ablate native tropism and incorporates a CD7‐targeting single‐chain variable fragment (scFv) as a membrane‐bound targeting moiety. This engineered configuration enables the precise delivery of a CAR20 transgene to T cells (comprising both CD4 and CD8 subsets) and NK cells, facilitating the generation of therapeutic CAR‐positive cells directly within the patient without requiring preconditioning lymphodepletion.

The first clinical proof‐of‐concept to evaluate safety, tolerability, and preliminary efficacy of in vivo CAR T‐cell therapy in humans is registered with ClinicalTrials.gov (NCT06691685) [95]. In a phase 1 dose‐escalation trial, four patients with relapsed or refractory multiple myeloma received a single intravenous infusion of ESO‐T01, a nanobody‐targeted and CD47‐shielded lentiviral vector designed to deliver an anti‐BCMA CAR transgene directly to endogenous T cells without prior apheresis or lymphodepleting chemotherapy. The treatment successfully generated functional, tumor‐infiltrating CAR T cells in vivo, leading to clinical responses, including stringent CRs in two heavily pretreated patients. While all patients experienced manageable CRS, no replicating virus was detected beyond 48 h, and toxicities were reversible with standard supportive care. This milestone demonstrates that targeted in vivo T‐cell engineering is clinically feasible, can bypass complex ex vivo manufacturing, and can induce antitumor activity in a challenging patient population, setting a foundational precedent for the field of in vivo CAR therapeutics.

#### 3.3.2 RNA‐Based Platforms

RNA‐based engineering platforms rely on linear mRNA or circular RNA to enable transient, non‐integrating CAR expression within immune cells. Early proof‐of‐concept studies utilized targeted nanoparticle, such as CD3‐ or CD8‐directed polymeric carriers loaded with anti‐CD19 CAR mRNA, to achieve in vivo T‐cell engineering and preclinical efficacy [121]. In recent years, LNPs have emerged as the leading formulation for systemic RNA delivery, owing to their established scalability from mass vaccination campaigns and continued innovation in ionizable lipid design, which together enhance tissue tropism, cellular uptake, and translational feasibility [122].

Hunter et al. report a transformative, non‐viral platform for generating functional CAR‐T cells directly in vivo [123]. The strategy employs CD8‐targeted lipid nanoparticles (tLNPs) to deliver anti‐CD19 chimeric antigen receptor (CAR) messenger RNA specifically to CD8^+^ T cells. The study provides compelling preclinical evidence across multiple models: the tLNP platform successfully reprogrammed T cells from both healthy donors and autoimmune patient samples ex vivo, controlled tumor growth in humanized mouse models, and achieved profound, transient depletion of B cells in the blood and tissues of cynomolgus monkeys. Notably, the reconstituted B cell compartment post‐depletion was predominantly naive, suggesting a potential “immune reset.” By circumventing the need for complex ex vivo cell manufacturing and lymphodepleting chemotherapy, this tLNP‐based approach presents a scalable, “off‐the‐shelf” alternative that could significantly broaden the accessibility and application of CAR‐T cell therapies for malignancies and autoimmune diseases.

#### 3.3.3 Key Regulators of CAR‐T Cell Fitness and Anti‐Tumor Efficacy

Two back‐to‐back *Nature* studies systematically applied CRISPR screening platforms to identify genetic modifiers that enhance CAR‐T cell function and persistence. Knudsen et al. performed an in vivo pooled loss‐of‐function CRISPR screen in BCMA‐targeting CAR‐T cells to identify genes that enhance their expansion and persistence in a human multiple myeloma xenograft model [124]. The “Mario” sgRNA library targeted 135 T cell–related genes. Screens were conducted at early (day 7) and late (day 21) time points in vivo, alongside in vitro manufacturing. The results revealed distinct gene requirements across contexts: RASA2 and SOCS1 ablation improved expansion in vitro, while loss of PTPN2, ZC3H12A, and RC3H1 conferred early in vivo advantages. Notably, CDKN1B (encoding the cell‐cycle inhibitor p27) emerged as the top hit limiting long‐term CAR‐T cell fitness in vivo. CDKN1B knockout enhanced CAR‐T cell proliferation, effector function, and tumor clearance, significantly prolonging survival in myeloma models. The study highlights that in vivo screens capture selective pressures missed in vitro and nominates CDKN1B ablation as a promising strategy to improve CAR‐T cell persistence in multiple myeloma.

In a complementary genome‐wide approach, the CELLFIE platform combined in vitro multiplexed functional screens with in vivo CROP‐seq validation in a leukemia model [125]. This study identified *RHOG* knockout as a potent and unexpected enhancer of CAR‐T cell efficacy, with synergistic effects when combined with *FAS* knockout. These findings were validated across donors, CAR designs, and patient‐derived cells, and further explored through combinatorial and base‐editing screens.

Together, these studies demonstrate the power of CRISPR screening and both focused in vivo and comprehensive in vitro platforms to uncover novel genetic targets that improve CAR‐T cell expansion, persistence, and tumor control. They highlight *CDKN1B* and *RHOG*/*FAS* as promising candidates for engineering next‐generation CAR‐T therapies with enhanced durability and efficacy.

### 3.4 Duchenne Muscular Dystrophy

Genome editing technology can repair gene mutations that cause genetic diseases, providing new hope for the treatment of genetic diseases such as Duchenne muscular dystrophy (DMD) [126, 127, 128, 129]. DMD is caused by mutations in the dystrophin gene that disrupt its reading frame, leading to a loss of functional protein. A promising therapeutic strategy aims to convert severe DMD into a milder Becker‐like phenotype by removing mutated exons to restore the reading frame. In a landmark study by Nelson et al. [126], this was achieved using a dual‐guide RNA (gRNA) approach, where two gRNAs targeted the introns flanking exon 23. Co‐delivery of Cas9 nuclease with these gRNAs generated DSBs in both introns, and subsequent repair via NHEJ resulted in the precise excision of exon 23, creating an in‐frame deletion resulting in partial recovery of functional dystrophin protein. For in vivo delivery, the researchers employed an adeno‐associated virus serotype 8 (AAV8) vector due to its tropism for muscle tissue. To overcome AAV's packaging limit (∼4.7 kb), they utilized the smaller SaCas9 and implemented a dual‐AAV system: one vector encoding SaCas9 and a second vector carrying the expression cassettes for the two gRNAs. Meanwhile, Long et al. [128] induced NHEJ by using a gRNA, SpCas9, and adeno‐associated virus serotype 9 (AAV9) for delivery to permanently delete the mutant exon 23 in the mdx mouse model after birth. Another study employed a streamlined sgRNA strategy targeting a critical sequence within the splice acceptor region of exon 51 in a canine model of DMD [127]. The mechanism involves Cas9 creating a DSB at this site, followed by error‐prone NHEJ repair. The repair process frequently introduces small insertions or deletions, most commonly a single adenosine (A) insertion in this case, which disrupts a key exonic splicing enhancer (ESE) in exon 51. Consequently, the splicing machinery fails to recognize the damaged exon, leading to its consistent exclusion during mRNA processing and ultimately restoring the dystrophin reading frame. Following systemic delivery of the CRISPR components via AAV9 in the canine model, this approach successfully restored dystrophin protein expression across a broad range of skeletal muscles and cardiac muscle.

### 3.5 CF

CF is caused by mutations in the *CFTR* gene, which encodes a chloride channel essential for fluid and electrolyte homeostasis across epithelial tissues, while the most common disease‐causing variant is a three‐base‐pair deletion leading to the loss of phenylalanine at position 508 (F508del) [130]. CRISPR/Cas9 genome editing system delivered into patient‑derived intestinal organoid cells via cationic lipid‑based transfection (Lipofectamine) is used to correct the CFTR locus by homologous recombination in CF patients‐derived cultured intestinal stem cells [131]. With a parallel comparative experimental design, two separate sets of experiments were conducted: one using a sgRNA targeting exon 11 of CFTR (sgRNA1), and the other using a sgRNA targeting intron 11 (sgRNA2). For both setups, a donor plasmid was constructed containing (1) the corrected F508del sequence, (2) a silent point mutation downstream to allow allele‑specific tracking, and (3) a puromycin‑resistance cassette inserted into the intron for selection. This parallel approach allowed the researchers to systematically compare the efficiency and outcomes of gene correction when the CRISPR‑Cas9‑induced double‑strand break was generated at different locations relative to the disease‑causing mutation. Recently, Geurts et al. [132] successfully corrected multiple nonsense mutations, including R785X, W1282X, R553X, and R1162X, in intestinal and upper airway organoids derived from CF patients using SpCas9 ABE and xCas9‐ABE. Restored CFTR was conformed to function with no detectable off‐target effects. This study provides the first experimental evidence for DSB‐free gene repair in CF patient‐derived organoids without genome‐wide off‐target effects. PE technology offers new hope for precisely correcting CFTR mutations, especially sites lacking NGG PAM sequences, such as F508del. Geurts et al. [133] demonstrated that PE could functionally repair the CFTR‐F508del mutation in patient‐derived intestinal organoids without detectable genome‐wide off‐target mutations but noted that its editing efficiency at this locus was notably low and was significantly outperformed by traditional HDR in a side‐by‐side comparison. Subsequent optimization efforts, however, have dramatically improved outcomes. Sousa et al. [134] systematically integrated six prime‐editing enhancements, including engineered pegRNAs (epegRNAs), the PEmax architecture, dominant‐negative MLH1 expression, strategic silent edits, evolved PE6 variants, and proximal “dead” sgRNAs to boost F508del correction efficiency from < 0.5% to 25% in primary airway epithelial cells from CF patients. This optimized prime‐editing strategy restored CFTR channel function to > 50% of wild‐type levels, comparable to the effect of small‐molecule modulator therapy, while maintaining a high edit‐to‐indel ratio and minimal off‐target editing. Schene et al. [135] developed a fluorescent PE and enrichment reporter (fluoPEER), providing a powerful tool for rapid screening and optimization of editing strategies targeting CFTR mutations. This system enables evaluation of the efficiency of different pegRNA combinations with multiple pre‐editor variants, such as SpG‐PE2 and SpRY‐PE2, which exhibit broader PAM recognition ranges. CFTR‐F508del and G542X (nonsense mutation) were efficiently repaired in patient‐derived intestinal organoids with fluoPEER.

### 3.6 p47^phox^‐Deficient Chronic Granulomatous Disease

Gori et al. report the first‐in‐human clinical investigation of PM359, an autologous CD34^+^ hematopoietic stem‐cell therapy that uses PE to correct the common two‐nucleotide deletion (delGT) in the NCF1 gene, which causes p47^phox^‐deficient chronic granulomatous disease (p47‐CGD) [94]. This study is registered with ClinicalTrials.gov (NCT06559176). Two adult male participants with p47‐CGD received a single intravenous infusion of PM359 following myeloablative conditioning with busulfan. The therapy involved electroporation of patient‐derived CD34^+^ cells with prime editor components to correct the delGT mutation. The study assessed engraftment, safety, and restoration of NADPH oxidase function in neutrophils using flow cytometry (dihydrorhodamine [DHR] assay) and DNA sequencing. Neutrophil and platelet engraftment occurred rapidly (within 14–19 days). Adverse events were consistent with busulfan conditioning, and no serious adverse events related to PM359 were reported. NADPH oxidase activity was restored within 1 month in both participants. Participant 1 (18 years old) achieved 69% DHR‐positive neutrophils by Day 30, with activity maintained through 6 months. Participant 2 (57 years old) achieved 83% DHR‐positive neutrophils by Day 30, with activity maintained through 4 months. The levels of functional correction significantly exceeded the 20% threshold associated with protection from severe infections. Participant 2 also showed a decrease in fecal calprotectin levels, indicating improvement in CGD‐associated colitis. The PE process resulted in high levels of on‐target correction with minimal unintended edits, and no evidence of off‐target editing or chromosomal abnormalities was detected in preclinical models. The study demonstrates that PE of autologous CD34^+^ cells is feasible at a clinical scale and can precisely correct the molecular defect in p47‐CGD. This resulted in the restoration of neutrophil function to near‐healthy levels in two participants, supporting the further development of PE as a therapeutic platform for genetic diseases.

### 3.7 Familial Hypercholesterolemia (heFH)

Building on the foundational mechanisms of ABE and CBE, recent studies have successfully translated these tools into promising in vivo therapies. VERVE‐101 is an investigational CRISPR base‐editing therapy designed to permanently inactivate the PCSK9 gene in the liver to achieve durable reductions in LDL‐cholesterol (LDL‐C). It was evaluated in the Phase Ib Heart‐1 trial (NCT05398029), an open‐label, single‐ascending dose study in patients with heterozygous familial heFH and established atherosclerotic cardiovascular disease (ASCVD) who were not at goal on maximal lipid‐lowering therapy. VERVE‐101 showed dose‐dependent LDL‐C reductions up to 55% at 0.6 mg/kg, sustained for 6 months in the Phase Ib Heart‐1 trial [88]. However, safety concerns emerged, including transient liver enzyme elevations and thrombocytopenia. One patient experienced a potential drug‐related myocardial infarction. Due to a serious adverse event involving ALT elevation and thrombocytopenia, enrollment was terminated. Development now focuses on VERVE‐102(NCT06164730), which uses the same base editor but a modified LNP delivery system (including GalNAc targeting) to improve safety [89].

In addition, Khirallah et al. reported a potential single‐treatment strategy for cardiovascular disease [136]. Using liver‐targeted LNPs to deliver an ABE8e system, they achieved durable knockout of the Angptl3 gene in mice via splice‐site editing, resulting in long‐term reductions of key atherogenic lipids. In parallel, VERVE‐201 employs N‐acetylgalactosamine (GalNAc)‐conjugated LNPs to deliver base editors specifically to hepatocytes in the liver, where they inactivate the ANGPTL3 gene to treat homozygous familial heFH (NCT06451770) [137, 138]. The GalNAc moiety enables targeted hepatic delivery by binding to the asialoglycoprotein receptor (ASGPR), which is highly expressed on hepatocytes but scarce in other tissues. This receptor‐mediated uptake is particularly advantageous for patients with homozygous familial heFH, who often have impaired low‐density lipoprotein receptor activity that can limit the effectiveness of unconjugated LNPs [137, 139].

### 3.8 Neonatal‐Onset Carbamoyl Phosphate Synthetase 1 Deficiency

Notably, the first personalized in vivo BE therapy was developed for a single patient with an ultra‐rare genetic disease [93]. The patient, diagnosed shortly after birth with neonatal‐onset carbamoyl phosphate synthetase 1 (CPS1) deficiency—a severe urea cycle disorder with high infant mortality—received a customized ABE delivered via LNPs to correct a disease‐causing nonsense variant (Q335X) in the CPS1 gene. The therapy, named k‐abe, was designed and manufactured within 6 months of the patient's birth. At approximately 7 and 8 months of age, the patient received two intravenous doses. Following treatment, the patient showed increased dietary protein tolerance, reduced need for nitrogen scavenger medication, and avoided hyperammonemic crises during viral infections—representing a significant clinical improvement. The study demonstrates the feasibility of rapidly developing and deploying patient‐specific, N‐of‐1 gene editing therapies for rare genetic conditions, highlighting the potential of platform‐based approaches using shared LNP and mRNA components with customized guide RNAs.

## 4 Delivery Systems: The Key to Clinical Translation

Delivery systems determine whether CRISPR components can reach target cells precisely, efficiently, and safely to exert their functions, and represent the common core bottleneck restricting the clinical translation of all CRISPR therapies. According to the location where editing occurs, CRISPR delivery can be divided into two major systems: ex vivo delivery and in vivo delivery. Ex vivo delivery involves isolating patient cells, editing them in vitro, and then infusing them back into the patient. It has the advantages of precise editing, controllable quality, and high safety, and is the mainstream technical route for all currently approved CRISPR therapies. In vivo delivery achieves in situ editing by directly administering drugs to patients, which has the potential for convenient operation, high accessibility, and no need for myeloablative conditioning, representing the development direction of next‐generation CRISPR therapies. This article systematically sorts out the technical characteristics of mainstream delivery platforms, focuses on the latest progress and challenges of in vivo delivery, and compares the clinical positioning and future trends of the two delivery systems.

### 4.1 Viral Vectors

Viral vectors have evolved over decades of gene therapy development and remain the most widely used delivery system for CRISPR‐based clinical applications due to their high transduction efficiency and ability to mediate long‐term transgene expression. The two most clinically relevant viral vectors are adeno‐associated virus (AAV) and lentivirus (LV), each with distinct technical characteristics and application scenarios.

#### 4.1.1 AAV

AAV is a non‐pathogenic, replication‐defective parvovirus that has become the preferred vector for in vivo CRISPR delivery due to its low immunogenicity, broad tissue tropism, and ability to mediate long‐term episomal expression in non‐dividing cells [140, 141]. More than 10 naturally occurring AAV serotypes and hundreds of engineered variants have been developed, each with preferential tropism for different tissues: AAV8 and AAV9 are highly efficient for liver and skeletal muscle transduction, AAV2 and AAV8 are widely used for retinal delivery, and AAV9 and AAVrh10 can cross the blood‐brain barrier to target the central nervous system [142].

The core clinical application of AAV is in vivo delivery for monogenic diseases affecting solid organs. For DMD, AAV‐mediated systemic delivery of CRISPR‐Cas9 components has been shown to restore dystrophin expression in skeletal and cardiac muscles in both mouse and canine models [126, 128]. In the field of ophthalmology, AAV‐mediated subretinal injection of CRISPR editors has achieved therapeutic levels of editing in preclinical models of Leber congenital amaurosis and other inherited retinal diseases [143]. For hemophilia B, AAV8‐delivered CRISPR base editors have demonstrated durable therapeutic efficacy in non‐human primates by correcting the disease‐causing mutation in the F9 gene.

Although AAV is primarily used for in vivo delivery, it also has important ex vivo applications. AAV vectors are increasingly being used for site‐specific gene integration in HSPCs and T cells, offering a safer alternative to lentiviral vectors due to their lower risk of insertional mutagenesis. For example, AAV6 has been shown to mediate efficient HDR in human HSPCs and CAR‐T cells, enabling precise insertion of therapeutic transgenes into safe harbor loci.

Despite its advantages, AAV has significant limitations. The most critical constraint is its small packaging capacity [142] (∼4.7 kb), which is insufficient to accommodate full‐length Cas9 and its guide RNA expression cassettes in a single vector. This limitation has driven the development of smaller Cas9 orthologs [144, 145, 146, 147] and compact Cas12‐family [148, 149] enzymes, as well as dual‐vector delivery strategies.

#### 4.1.2 LV

LVs are a subclass of retroviruses that can transduce both dividing and non‐dividing cells and integrate their genome into the host chromosome, enabling stable long‐term expression of transgenes. LV has become the gold standard for ex vivo CRISPR delivery, particularly for CAR‐T cell engineering and HSPC editing, due to its large packaging capacity (∼8 kb) and high transduction efficiency in hematopoietic cells.

All currently approved CAR‐T cell therapies use LV vectors to deliver the CAR transgene into autologous T cells [112, 113, 114]. In CRISPR‐engineered allogeneic CAR‐T cells, LV vectors are used in combination with CRISPR nucleases to simultaneously deliver the CAR transgene and disrupt genes associated with GVHD and immune rejection (e.g., TRAC, B2M, and PD1).

In recent years, significant progress has been made in developing LV vectors for in vivo CRISPR delivery, particularly for in vivo CAR‐T cell therapy. The VivoVec platform uses engineered LV vectors displaying a multidomain fusion protein that provides integrated T‐cell activation and costimulatory signals, enabling efficient generation of anti‐CD20 CAR‐T cells in non‐human primates with sustained B‐cell depletion for more than 10 weeks [120]. INT2104, a clinical candidate using this platform, utilizes a detargeted viral fusogen and a CD7‐targeting scFv to specifically deliver a CAR20 transgene to T cells and NK cells in vivo without the need for preconditioning lymphodepletion [120]. Most notably, the first‐in‐human clinical trial of in vivo CAR‐T therapy (ESO‐T01) used a nanobody‐targeted LV vector to deliver an anti‐BCMA CAR transgene directly to endogenous T cells in patients with relapsed or refractory multiple myeloma, resulting in stringent CRs in two heavily pretreated patients [95].

The main limitation of LV vectors is their potential for random genomic integration, which carries a risk of insertional mutagenesis and oncogene activation. Although modern self‐inactivating (SIN) LV vectors have significantly reduced this risk, it remains a safety concern for long‐term clinical applications. Other challenges include the high cost and complexity of large‐scale LV production, as well as pre‐existing immunity to LV envelope proteins in some patients.

### 4.2 Non‐Viral Platforms

Non‐viral delivery platforms have emerged as promising alternatives to viral vectors due to their lower immunogenicity, reduced risk of genomic integration, unlimited packaging capacity, and scalable manufacturing. The most advanced non‐viral platforms for CRISPR delivery include LNPs, virus‐like particles (VLPs), and polymeric nanoparticles.

#### 4.2.1 LNPs

LNPs are the most clinically advanced non‐viral delivery platform, with proven safety and efficacy in mRNA vaccines for COVID‐19. They are composed of ionizable lipids, helper lipids, cholesterol, and polyethylene glycol (PEG)‐lipids, which self‐assemble into nanoparticles that encapsulate and protect nucleic acids from degradation and facilitate cellular uptake via endocytosis.

The core clinical application of LNPs for CRISPR delivery is in vivo liver‐targeted delivery, as LNPs naturally accumulate in the liver following intravenous administration. VERVE‐101, the first in vivo CRISPR BE therapy to enter clinical trials, uses LNPs to deliver an ABE targeting the PCSK9 gene in hepatocytes for the treatment of heterozygous familial heFH [88]. Although enrollment in the VERVE‐101 trial was terminated due to safety concerns, the next‐generation therapies VERVE‐102 [89] and VERVE‐201 [90] use GalNAc‐conjugated LNPs to enhance hepatocyte‐specific delivery and improve the safety profile. The first personalized in vivo BE therapy, k‐abe, also uses LNPs to deliver a customized ABE to correct a disease‐causing nonsense variant in the CPS1 gene in a patient with neonatal‐onset urea cycle disorder [93].

In addition to in vivo delivery, LNPs are also used for ex vivo delivery of CRISPR mRNA and RNP complexes into HSPCs and T cells. For example, LNP‐168, an antibody‐free LNP platform, enables efficient in vivo editing of the HBG1/2 promoter in human HSPCs to reactivate HbF, offering a potential non‐myeloablative treatment for β‐thalassemia [103].

Recent advances in LNP engineering have focused on expanding tissue tropism beyond the liver. Ionizable lipids with altered physicochemical properties have been developed to target the spleen, lungs, and muscles. GalNAc conjugation has become a standard strategy for enhancing liver targeting, as GalNAc binds specifically to the ASGPR highly expressed on hepatocytes. Other targeting strategies include the conjugation of antibodies or aptamers to the LNP surface to direct delivery to specific cell types.

#### 4.2.2 VLPs

VLPs are non‐infectious, self‐assembling protein nanoparticles that mimic the structure of viruses but lack viral genetic material. They combine the advantages of viral vectors (high transduction efficiency) and non‐viral platforms (no risk of genomic integration, transient expression), making them ideal for delivering CRISPR RNPs.

Engineered virus‐like particles for prime editing (PE‐eVLPs) represent a major breakthrough in VLP‐mediated CRISPR delivery. Through systematic optimization of the VLP architecture, including protease site engineering, nuclear export signal relocation, and incorporation of an MCP‐MS2 system for enhanced pegRNA packaging, PE‐eVLPs achieve 65‐ to 170‐fold higher PE efficiency in human cells compared to early versions. In two mouse models of inherited retinal degeneration, a single subretinal injection of v3 PE‐eVLPs mediated therapeutic levels of PE, restored target protein expression, and partially rescued visual function. Importantly, transient delivery as RNPs reduces off‐target editing and eliminates risks associated with viral DNA integration [120].

The main limitations of VLPs are their relatively small packaging capacity and the complexity of large‐scale production. However, ongoing engineering efforts are addressing these challenges, and VLPs are expected to play an increasingly important role in in vivo CRISPR delivery for a wide range of diseases.

#### 4.2.3 Polymeric and Inorganic Nanoparticles

Polymeric nanoparticles, composed of biodegradable polymers such as polyethylenimine (PEI), chitosan, and poly(lactic‐co‐glycolic acid) (PLGA), offer high stability, low cost, and versatile surface modification capabilities. They have been used to deliver CRISPR components to various cell types in vitro and in vivo, particularly for local delivery to tumors. Inorganic nanoparticles, such as gold nanoparticles and magnetic nanoparticles, have also been explored for CRISPR delivery due to their unique optical and magnetic properties, which enable targeted delivery and imaging.

However, polymeric and inorganic nanoparticles generally have lower transduction efficiency compared to LNPs and viral vectors, and their in vivo clearance is relatively fast. Further optimization is needed to improve their efficiency and biocompatibility for clinical translation.

### 4.3 Tissue‐Specific Delivery Strategies

The ability to deliver CRISPR components specifically to target tissues while avoiding off‐target editing in healthy tissues is critical for the safety and efficacy of in vivo CRISPR therapies. Significant progress has been made in developing tissue‐specific delivery strategies for the most clinically relevant organs.

#### 4.3.1 Liver‐Targeted Delivery

The liver is the most accessible organ for systemic delivery, and liver‐targeted CRISPR therapies are the most advanced in clinical development. Two main strategies are used for liver targeting: GalNAc‐conjugated LNPs and liver‐tropic AAV serotypes (AAV8, AAV9). GalNAc‐LNPs have become the gold standard for liver delivery of mRNA and base editors, as they mediate highly specific uptake by hepatocytes via ASGPR binding. This strategy is used in VERVE‐102 [89], VERVE‐201 [90], and several other clinical‐stage liver‐targeted CRISPR therapies.

#### 4.3.2 Ocular Delivery

The eye is an ideal organ for local CRISPR delivery due to its immune‐privileged status and easy accessibility. Subretinal injection and intravitreal injection are the two main routes of ocular delivery. Subretinal injection of AAV vectors has been used successfully in preclinical models of inherited retinal diseases, and several clinical trials are currently underway [76].

#### 4.3.3 Hematopoietic System Delivery

In vivo delivery to HSPCs in the bone marrow would eliminate the need for ex vivo cell manipulation and myeloablative conditioning, significantly improving the accessibility of CRISPR therapies for blood disorders. Recent advances include the development of CD34‐targeted antibody‐conjugated LNPs and HSPC‐tropic AAV variants. LNP‐168, as mentioned earlier, enables efficient in vivo editing of human HSPCs in a humanized mouse model, representing a major step toward in vivo HSPC editing [103].

#### 4.3.4 Muscle‐Targeted Delivery

Systemic delivery of AAV9 vectors is the most advanced strategy for muscle‐targeted CRISPR delivery for DMD and other muscular dystrophies [126]. AAV9 has a natural tropism for skeletal and cardiac muscles, and systemic administration results in widespread muscle transduction. However, the high doses required for systemic muscle delivery increase the risk of immune responses and liver toxicity, highlighting the need for more efficient and specific muscle‐targeted delivery systems.

## 5 Challenges and Future Perspective

### 5.1 Off‐Target Activity

Numerous studies report that Cas9 can bind and cleave partially complementary off‐target sequences, ranging from a single base mismatch to multiple consecutive mismatches, or insertions or deletions [150, 151, 152, 153]. The risks of multiplexed off‐target editing by Cas9 include genomic rearrangement, such as deletions, inversions, and chromosomal translocations, which can subsequently activate DNA damage and cellular stress response pathways [154, 155], therefore off‐target activity remains a safety concern for clinical applications. Chromosomal loss is a significant safety concern requiring urgent attention in CAR‐T cell therapies [155]. A systematic study by Tsuchida et al. [155] revealed that CRISPR‐Cas9 editing of T cells leads to partial or complete loss of the targeted chromosome across the genome and occurs even during the preclinical preparation of CAR‐T cells. Notably, by optimizing the preparation workflow, Tsuchida et al. [155] adopted an “edit‐then‐activate” strategy in the first‐in‐human clinical trial (NCT03399448). This modification significantly reduced the incidence of chromosomal loss while largely preserving gene editing efficiency, offering a practical solution for enhancing the safety of clinical‐grade engineered T‐cell products.

To mitigate off‐target risks, researchers have developed high‐fidelity Cas9 variants such as eSpCas9(1.1) [156], Cas9–HF1 [157], HypaCas9 [158], Cas9_R63A/Q768A [159], evoCas9 [160], HiFi Cas9 [161], and Sniper2L [162] through two strategies. First, fidelity‐enhancing mutations were designed based on structure, where fidelity‐enhancing mutations weaken Cas9's non‐specific binding to non‐target DNA, rendering it more sensitive to sequence mismatches. The underlying principle is to promote dissociation at off‐target sites by slowing the cleavage rate [156, 162]. Second, directed evolution is employed to directly screen for highly specific variants [160, 163]. These variants (e.g., HypaCas9 and evoCas9) significantly enhance editing specificity, though their efficiency may vary by different target DNA sites [164, 165]. Moreover, they cannot entirely eliminate all off‐target possibilities, as each gRNA exhibits a unique off‐target profile. To mitigate off‐target risks, various in silico tools have been developed to design sgRNAs. In addition to conventional hypothesis‐driven (e.g., CRISPRoff [166], uCRISPR [167], MIT [168], and CFD [169]) and learning‐based approaches (e.g., deepCRISPR [170] and DL‐CRISPR [171]), emerging strategies integrate molecular dynamics (MD) simulations with machine learning to capture RNA–DNA interaction fingerprints for enhanced off‑target prediction and sgRNA optimization [172].

Using truncated guide RNAs for Cas9, where the guide segment is reduced from 20 to 17–18 nucleotides, significantly reduces the off‐target activity of SpCas9 at various sites [151, 173], but can also result in decreased efficiency or new off‐target sites [156]. Modifications to the 5′ end of the guide segment, either through the introduction of secondary structures [174] or unpaired nucleotides [175], can also reduce the off‐target activity. Evidence confirm the functionality of “hybrid” RNA‐DNA guides and demonstrate that 2′‐deoxynucleotide substitutions within the guide segment significantly boost gene editing specificity in cells [176, 177, 178, 179].

The clinical deployment of CRISPR‐engineered cell therapies, while promising, is accompanied by a range of scientific, technical challenges that must be addressed to ensure safe and effective translation. One of the foremost concerns is the genomic safety of BE and PE. Although these technologies avoid DSBs and reduce the risk of chromosomal translocations, they are not entirely free from off‐target effects. Studies have shown that certain base editors can induce guide RNA‐dependent and independent off‐target edits, including bystander mutations and unintended deamination of RNA and DNA. Therefore, rigorous genotoxicity assessments using sensitive assays such as GUIDE‐seq [151], CAST‐seq [180], or CHANGE‐seq [181], followed by validation in primary human T cells, are essential prior to clinical application. Moreover, the functional consequences of bona fide off‐target edits remain difficult to predict and require long‐term monitoring of edited clones in vivo. Additionally, single‐strand nicks can occasionally resolve into DSBs, posing a potential genotoxic risk [182]. In response, ongoing protein engineering efforts have substantially enhanced the precision and efficiency of base editors, resulting in next‐generation CBEs and ABEs featuring optimized architectures, evolved deaminase variants, and markedly reduced RNA off‐target activity [183, 184, 185, 186].

### 5.2 Immune Response

Beyond off‐target effects and delivery efficiency, host immune responses have emerged as another critical barrier to the clinical translation of CRISPR‐Cas9. Due to the origin of Cas9 proteins from common pathogens such as SaCas9 (*Staphylococcus aureus*) and SpCas9 (*Streptococcus pyogenes*), extensive pre‐existing adaptive immunity exists within the human body [187, 188] and Cas9‐specific immune responses were reported to compromise outcomes of CRISPR therapy in dystrophic canine models [189]. Pre‐existing adaptive immunity poses two threats. First, humoral immunogenicity can lead to the rapid clearance of viral vectors (e.g., AAV) or Cas9 protein, reducing delivery efficiency and editing efficacy. Second, cellular responses may trigger cytotoxic T lymphocyte (CTL) attacks against successfully edited cells that present Cas9 peptides on their MHC‐I, potentially eliminating the therapeutic cell population and causing inflammatory toxicity. To overcome re‐existing adaptive immunity, reduced immunogenicity Cas variants of SaCas9, AsCas12a [190], and SpCas9 [191] have been designed by eliminating immunogenic epitopes with targeted mutation.

From a therapeutic perspective, selecting the optimal combination of gene edits to enhance T‐cell function without inducing hyper‐reactivity or autoimmunity is a complex task. The simultaneous multiplex editing enabled by CRISPR increases the risk of unintended phenotypic shifts, such as altered differentiation states or off‐tumor toxicity. Integrating safety switches, such as inducible suicide genes, and employing strategies to tune receptor affinity or disrupt antigen presentation in healthy tissues will be essential to mitigate these risks [192]. Furthermore, the transition from autologous to allogeneic T‐cell products introduces additional hurdles, including immune rejection and GVHD, which require sophisticated multiplex editing strategies that must be balanced with genomic stability [193].

### 5.3 Editing Efficiency

Another significant challenge lies in the variability and efficiency of editing, particularly with PE. While BE can achieve > 98% editing efficiency in primary T cells, PE often exhibits variable efficiency across different target sites, and its reliance on complex prime editing guide RNA (pegRNA) design can hinder widespread adoption. However, recent advances in machine learning‐based pegRNA design tools and high‐throughput screening platforms are beginning to address these limitations. For BE, the limited types of editable nucleotides and the presence of bystander edits remain constraints, although the development of transversion base editors and high‐fidelity variants is expanding the toolkit. Continued protein engineering and optimization of delivery methods, such as RNP complexes or LNPs, will be critical to improving the precision and scalability of CRISPR technologies.

### 5.4 Regulatory and Ethical Barriers

Beyond technical delivery and safety challenges, the widespread clinical translation of CRISPR‐based therapies faces significant regulatory and ethical barriers. Global regulatory bodies have adopted divergent frameworks for approving genome‐edited products: while the FDA and EMA have granted accelerated designations to ex vivo HSC therapies, standardized long‐term safety monitoring protocols for novel modalities such as in vivo BE and PE remain under development, creating uncertainty for clinical development pathways. An established global ethical consensus prohibits heritable germline genome editing for human reproduction due to its irreversible impact on the human gene pool and inherent violation of intergenerational informed consent [194]. For somatic CRISPR therapies, the most pressing ethical issue is equitable access: the high manufacturing costs and complex personalized workflows result in million‐dollar price points for approved products, putting curative treatment out of reach for the vast majority of patients worldwide, particularly in low‐ and middle‐income regions with the highest burden of hemoglobinopathies [195]. Additional concerns include adequate informed consent for patients with rare genetic diseases, long‐term pharmacovigilance of potential delayed adverse events, and guardrails against non‐therapeutic genetic enhancement, all of which require coordinated international governance to ensure responsible innovation.

## 6 Conclusion

CRISPR technology has grown from a prokaryotic adaptive immune mechanism into a versatile genome engineering platform that is revolutionizing basic biological research and clinical medicine. This review has systematically delineated the expanding CRISPR toolbox—encompassing canonical Cas9 nucleases, precision base editors, prime editors, CRISPR‐associated transposases, transcriptional regulation systems, and Cas12/Cas13 RNA‐targeting tools—with detailed discussion of their molecular mechanisms, inherent advantages, and remaining limitations. We further summarized the rapidly advancing clinical landscape of CRISPR‐based therapies, from the first approved ex vivo editing therapy for hemoglobinopathies to emerging clinical candidates leveraging BE, PE, and in vivo CAR‐T engineering for a wide range of hematological, oncological, cardiometabolic, and rare genetic diseases. The critical role of delivery systems, including viral vectors, LNPs, and tissue‐specific targeting strategies, in enabling clinical translation was also emphasized.

Despite remarkable progress, several fundamental challenges continue to limit the broader application of CRISPR therapeutics, including off‐target genotoxicity, pre‐existing immunogenicity of bacterial‐derived Cas proteins, variable editing efficiency in primary cells, and the lack of universal tissue‐specific in vivo delivery systems, alongside regulatory and ethical barriers surrounding equitable access and long‐term safety. Ongoing advances in high‐fidelity effector engineering, immune epitope silencing, and next‐generation delivery technologies are progressively addressing these obstacles. Looking ahead, continued synergistic innovation in editing tools, delivery platforms, and clinical trial design will further expand the therapeutic scope of CRISPR medicine, cementing its position as a cornerstone of next‐generation precision medicine and offering curative hope for patients with currently intractable diseases.

## Author Contributions

Xinzhi Ma and Yingli Sun contributed to the writing, illustration, and revision of the article. Lingyu Li searched for reference papers. All authors reviewed and approved the final manuscript.

## Conflicts of Interest

The authors declare no conflicts of interest.

## Ethics Statement

The authors have nothing to report.

## Data Availability

The authors have nothing to report.
